# A moso bamboo *WRKY* gene *PeWRKY83* confers salinity tolerance in transgenic Arabidopsis plants

**DOI:** 10.1038/s41598-017-10795-z

**Published:** 2017-09-15

**Authors:** Min Wu, Huanlong Liu, Guomin Han, Ronghao Cai, Feng Pan, Yan Xiang

**Affiliations:** 1 National Engineering Laboratory of Crop Stresses Resistance Breeding, Anhui Agricultural University, Hefei, 230036 China; 2 Laboratory of Modern Biotechnology, School of Forestry and Landscape Architecture, Anhui Agricultural University, Hefei, 230036 China

## Abstract

The *WRKY* family are transcription factors, involved in plant development, and response to biotic and abiotic stresses. Moso bamboo is an important bamboo that has high ecological, economic and cultural value and is widely distributed in the south of China. In this study, we performed a genome-wide identification of *WRKY* members in moso bamboo and identified 89 members. By comparative analysis in six grass genomes, we found the *WRKY* gene family may have experienced or be experiencing purifying selection. Based on relative expression levels among *WRKY* IIc members under three abiotic stresses, *PeWRKY83* functioned as a transcription factor and was selected for detailed analysis. The transgenic Arabidopsis of *PeWRKY83* showed superior physiological properties compared with the WT under salt stress. Overexpression plants were less sensitive to ABA at both germination and postgermination stages and accumulated more endogenous ABA under salt stress conditions. Further studies demonstrated that overexpression of *PeWRKY83* could regulate the expression of some ABA biosynthesis genes (*AtAAO3*, *AtNCED2*, *AtNCED3*), signaling genes (*AtABI1*, *AtPP2CA*) and responsive genes (*AtRD29A*, *AtRD29B*, *AtABF1*) under salt stress. Together, these results suggested that *PeWRKY83* functions as a novel *WRKY*-related TF which plays a positive role in salt tolerance by regulating stress-induced ABA synthesis.

## Introduction

Grasses (Poaceae) are one of the most important plant families; they are not only the major source of food and potential renewable energy, but have huge economic and ecological value^1^. The grasses can be divided into several economically important subfamilies based on genomic analyses, such as the Bambusoideae (moso bamboo), Panicoideae (maize and sorghum), Ehrhartoideae (rice) and Pooideae (*Brachypodium*). Every year, the geographical distribution, growth, development and yield of many grass plants are limited by extreme environmental conditions, such as drought, high salinity or cold temperature^2^. Moso bamboo (*Phyllostachys edulis*), one of the most important woody bamboo, represents the only Bambusoideae plant. China is the most important area for moso bamboo, with many natural *Phyllostachys edulis* forests. However, in the south of China, unfavourable conditions (salinity and soil depletion) and extreme climate (drought and cold) limit the growth and distribution of moso bamboo.

Transcription factors (TFs) play a key role in plant growth and development, and respond to biotic and abiotic stresses through interaction with *cis*-acting elements in promoter regions, or with other TFs to regulate gene expression. Currently, there are more than 60 TF families identified with different functional roles^3^. The *WRKY* gene family is the largest TF family in plants. The *WRKY* TFs are named as such due to the conserved *WRKY*GQK sequence in the N-terminal, followed by a zinc finger motif^4^. In some *WRKY* genes, the *WRKY* domain is replaced by WKKY, WKRY, WSKY, WIKY, WRIC, WRMC, WRRY or WVKY^5,6^. There are three types of *WRKY* proteins in the *WRKY* gene family: group I-III. The classification is based on the number of *WRKY* domains and the pattern of the zinc finger motif. Proteins which contain two *WRKY* domains including a C_2_H_2_ (C-X_4-5_-C-X_22-23_-H-X-H) motif belong to group I. Group II and Group III proteins have one *WRKY* domain, but different finger motifs. Group II contains the same zinc-finger motif (C_2_H_2_) as group I. Group III has a C_2_-HC (C-X_5-8_-C-X_25-28_-H-X_1-2_-C) motif^4^. Most proteins with one *WRKY* domain belong to group II, and group II is further classified into five subgroups (IIa, IIb, IIc, IId, and IIe) based on their phylogenetic clades^4^. It is well known that *WRKY* proteins promote the expression of downstream target genes, since they can specifically interact with the W-box ((C/T)TGAC[T/C]) or the SURE (sugar-responsive *cis*-element) in the promoter region of many plant target genes^7,8^.

In 1994, the first *WRKY* protein (SPF1) was cloned from sweet potato by Ishiguro and Nakamura *et al*.^9^. Subsequently, many *WRKY* protein genes were cloned from different plant species especially in grass plants: rice, maize, *Brachypodium distachyon*, wheat and barley^10–14^. To date, only two *WRKY* homologues have been identified in non-plant species, *Giardia lamblia* and *Dictyostelium discoideum* ^15,16^. Moreover, the functions of *WRKY* proteins have been discovered. It has been shown that *WRKY* proteins play key roles in plant response to bacterial, fungal and viral pathogens in Arabidopsis, rice, tobacco, and parsley^17–19^. Furthermore, there is evidence that *WRKY* proteins are involved in response to various abiotic stresses, such as high temperature, low temperature, salt, drought, and ABA. In Arabidopsis, *WRKY25*, *−26* and *−33* play significant roles in response to heat tolerance^20^ and *AtWRKY57* can improve drought stress^21^. There were at least nine *WRKY* genes in soybean which are differentially expressed under abiotic stresses^22^. The transgenic plants of *GmWRKY21* and *−13* were tolerant to cold and salt stresses, respectively. *GmWRKY54* was involved in drought and salt tolerance^22^. In cotton, resistance to salt and drought of *GhWRKY68* was reduced, when overexpressed in *Nicotiana benthamiana* ^23^. The function of *WRKY* proteins have been studied in grass plants. It is well documented that *WRKY* proteins are involved in the regulation of plant growth and developmental processes, including trichome development^24^, seed development and germination, embryogenesis and leaf senescence^25–27^.

The functional knowledge of *WRKY* proteins has been widely studied. The aim of this study was to identify whether *PeWRKY* proteins have a common function. The moso bamboo whole genome has been sequenced^28^; therefore a genome-wide analysis of the *WRKY* gene family in moso bamboo could be performed. The aim of our study was to characterize the *WRKY* protein family of moso bamboo, by identifying their protein sequence characteristics, phylogenetic relationships and gene structures. Moreover, *PeWRKY83* was selected for detailed functional analysis.

## Results

### Identification and classification of *PeWRKY* genes

Based on the HHM profile of the *WRKY* domain (PF03106), 102 putative *WRKY* genes were identified in the moso bamboo genome (http://www.ncgr.ac.cn/bamboo, accessed February 2016). These candidates were subjected to Interproscan program to confirm the presence of the *WRKY* domains. Lastly, we obtained 89 protein-coding genes in moso bamboo. The 89 *WRKY* genes from moso bamboo are uniformly named as *PeWRKY1*-*PeWRKY89*, according to their physical locations (from top to bottom) on the chromosomes (Table S1).

**Figure 1:**
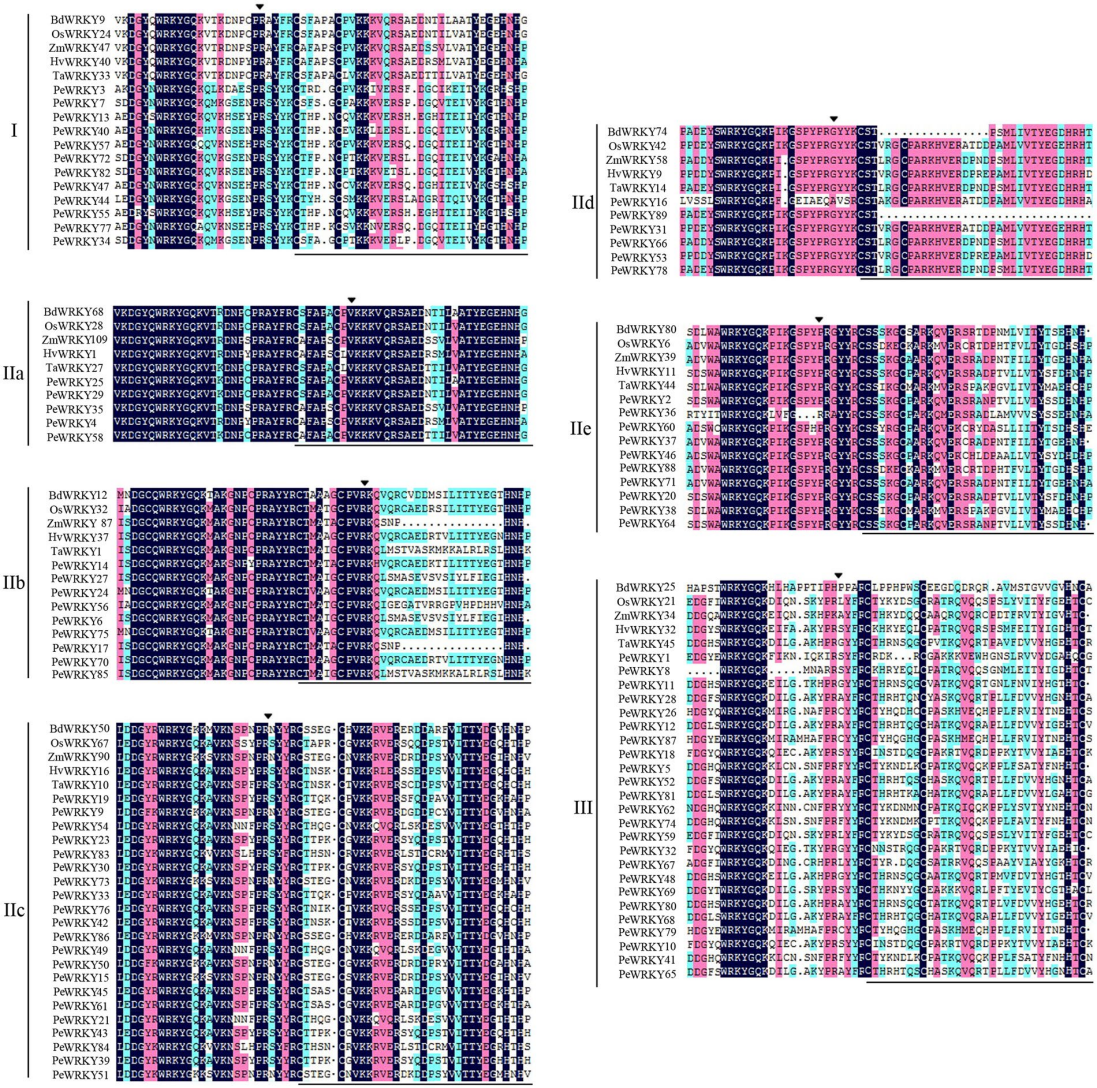
Alignment of multiple *PeWRKY* and selected other Gramineae *WRKY* domain amino acid sequences. Alignment was performed using the DNAMAN software. Residues that were highly conserved within each of the major groups are indark blue. The position of a conserved intron was indicated by an arrowhead. The black lines indicated the conserved zinc finger motifs.

The phylogenetic relationship of the *PeWRKY* proteins was examined by multiple sequence alignment of their *WRKY* domains, which span approximately 60 amino acids (Fig. 1). The comparison analysis of the *WRKY* domains from six different grasses *WRKY* proteins, resulting in a better separation of the different groups and subgroups. For each of the groups or subgroups, I, IIa to IIe and III, one representative from *Brachypodium distachyon*, rice, maize, wheat and barley was chosen randomly. As shown in Fig. 1, the sequences of the *WRKY* domains were highly conserved. The highly conserved *WRKY* motif, including 80 *WRKY*GQK, seven *WRKY*GKK, one *WRKY*GQA and one *WRKY*GQQ existed in the 89 *PeWRKY* genes. In the 89 *PeWRKYs*, 13 of the *WRKY* proteins contained two complete *WRKY* domains and a C_2_-H_2_ type zinc finger motif. These proteins constituted group I. 76 *WRKY* proteins contained one *WRKY* domain, of which 51 (67%) have a C_2_H_2_-type (C-X_4-5_-C-X_22-24_-H-X_1-2_-H) zinc finger motif belonging to group II and the remaining 25 (33%) *WRKY* proteins belong to group III, which had a C_2_-HC type (C-X_6-7_-C-X_23-33_-H-X_1_-C) zinc finger motif. Furthermore, group II proteins can be divided into five distinct subgroups (2a-e), there were 5, 9, 21, 6 and 10 members in subgroups IIa-e, respectively.

The conserved motifs of *PeWRKY* proteins were further identified using MEME program. A total of 20 conserved motifs were identified. The different motifs were identified based on the biochemical properties of their amino acids and their specific location in the protein sequence (Figure S1)^12^. The conserved amino acids, the position of each residue in the *WRKY* sequence, and the residue varied greatly (Figure S1, Table S2). The conserved motifs 2, 4, 8 and 18 encoded the *WRKY* domains, which were broadly distributed in the *PeWRKY* protein sequences. From the 13 members of group I, two *WRKY* domains (motif 2 and motif 4) were identified, while in the other members from group II or III, there was only one *WRKY* domain. Most *PeWRKY* members from the same group, especially the closely related members, shared common motif compositions, while only seven motifs (motif 6, 8, 10, 15, 17, 18 and 19) were shared by different groups. These results suggest that the most prominent features of *PeWRKY* are highly conserved.

### Phylogenetic analysis of *PeWRKYs* in six grass genomes

**Figure 2:**
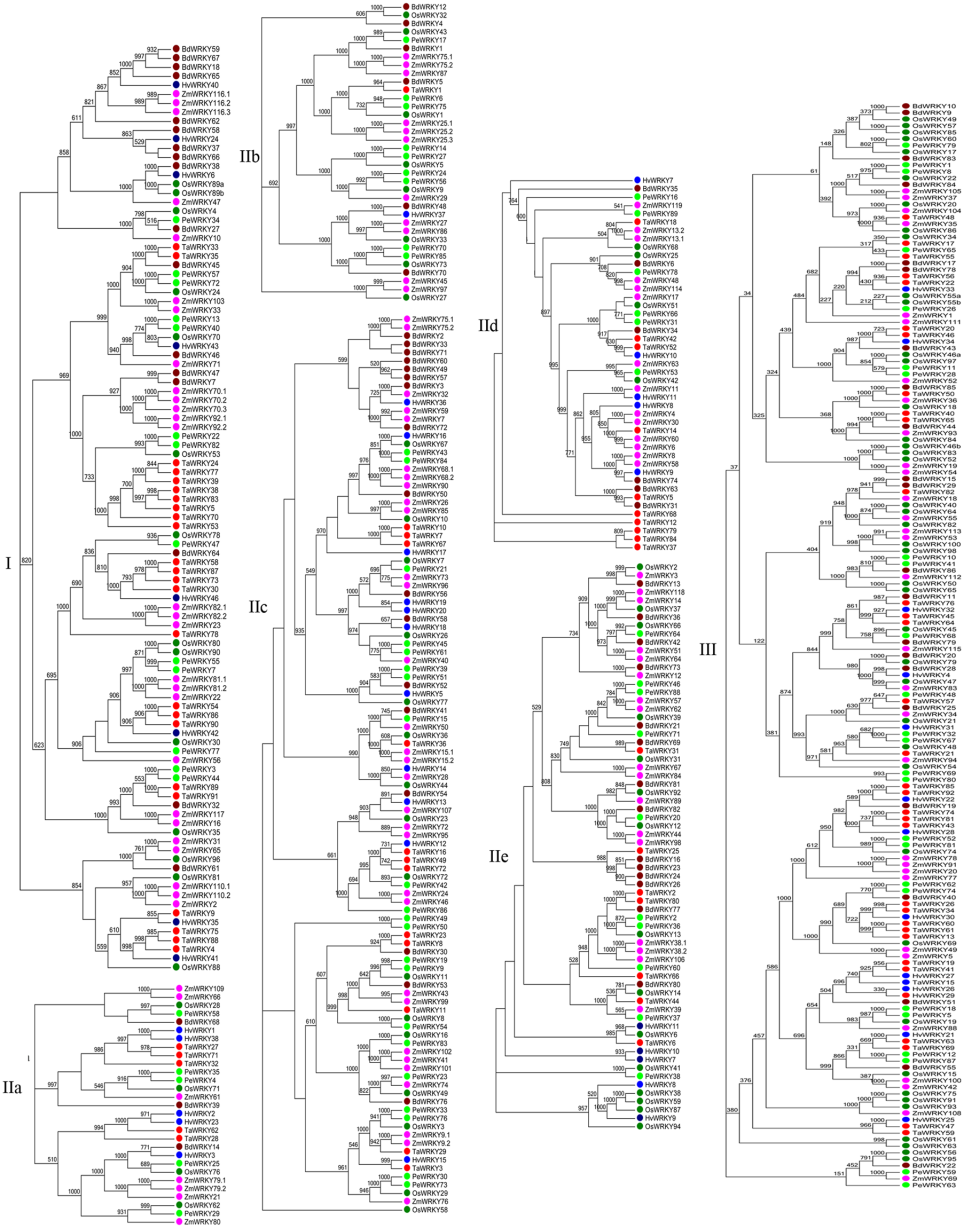
Phylogenetic tree of *WRKY* proteins of moso bamboo, *Brachypodium distachyon*, rice, maize, wheat and barley. The tree was generated using MEGA 6.0 by the NJ method with 1,000 bootstrap replicates. Moso bamboo, *Brachypodium distachyon*, rice, maize, wheat and barley proteins are indicated with different-coloured dots.

To examine the phylogenetic relationship among all 89 *PeWRKY* domains, a phylogenetic tree based on conserved *WRKY* domains containing *Brachypodium distachyon*, rice, maize, wheat and barley was constructed. Two different phylogenetic trees were created (Neighbour-Joining and Maximum Parsimony) using MEGA 6.0. The two kind of phylogenetics were largely comparable with only minor modifications at interior branches (data not shown). Therefore, the N-J phylogenetic tree was classified into three well-conserved subfamilies, group I to III (Fig. 2), as described previously and with significant statistical support^10,11,13,14,29^. This complex phylogenetic tree displayed the classification of *PeWRKYs* based on multiple sequence alignment of their *WRKY* domains, and the phylogenetic relationship of *PeWRKYs* was conserved (Figure S2).

**Figure 3:**
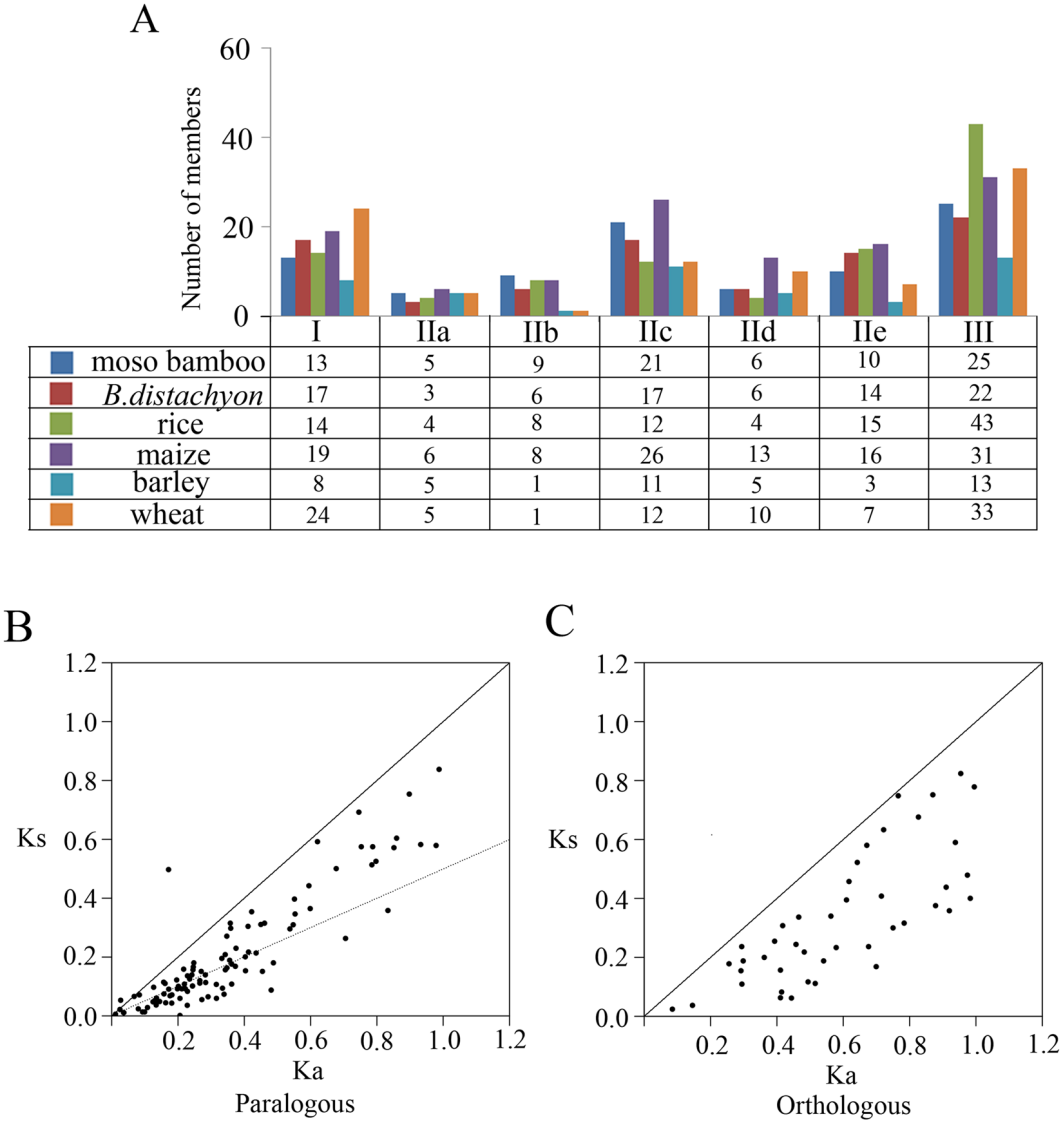
Comparison of *WRKY* family among six grass species and distribution of Ka and Ks values. (**A**) Comparison of group/subgroup size among moso bamboo, *Brachypodium distachyon*, *Oryza sativa*, *Zea maize*, *Triticum aestivum* and *Hordeum vulgare WRKY* family. (**B**) Above the black line, paralogous pairs with Ka/Ks ratio >1; between solid and dashed lines, pairs with Ka/Ks ratio 0.5–1. (**C**) Above the black line, orthologous pairs with Ka/Ks ratio >1; between solid and dashed lines, pairs with Ka/Ks ratio 0.5–1.

The number of *WRKY* proteins from each subfamily among six plant species was listed (Fig. 3A), revealing group II was the largest subfamily of the six-plant species. This was supported by that previously report-most proteins with one *WRKY* domain belong to group II. To clarify the paralogous and orthologous relationships among this family, we used two popular methods: phylogeny-based and bidirectional best-hit. Tables S3 details 104 putative paralogous pairs and 43 putative orthologous The Ka/Ks ratios of all the paralogous pairs ranged from 0.0078 to 0.974 and the mean value approximately 0.513 (Fig. 3B), while the Ka/Ks ratios of all 43 putative orthologous pairs ranged from 0.139 to 0.977 and the mean value approximately 0.517 (Fig. 3C). The Ka/Ks ratios of all the *WRKY* paralogous and orthologous pairs were less than 1, representing purifying selection on the *WRKY* genes.

### Expression profile of *PeWRKY* IIc genes

Numerous experiments in grasses have showed that *WRKY* IIc genes can be involved in various physiological processes under normal growth conditions and under various stresses. The sequence and evolutionary relationship analyses identified 21 *PeWRKY* genes in the group IIc. Thereafter, ten candidate *PeWRKY* IIc genes were selected and their expression levels in response to abiotic stress by quantitative real-time PCR (qRT-PCR) were performed.

**Figure 4:**
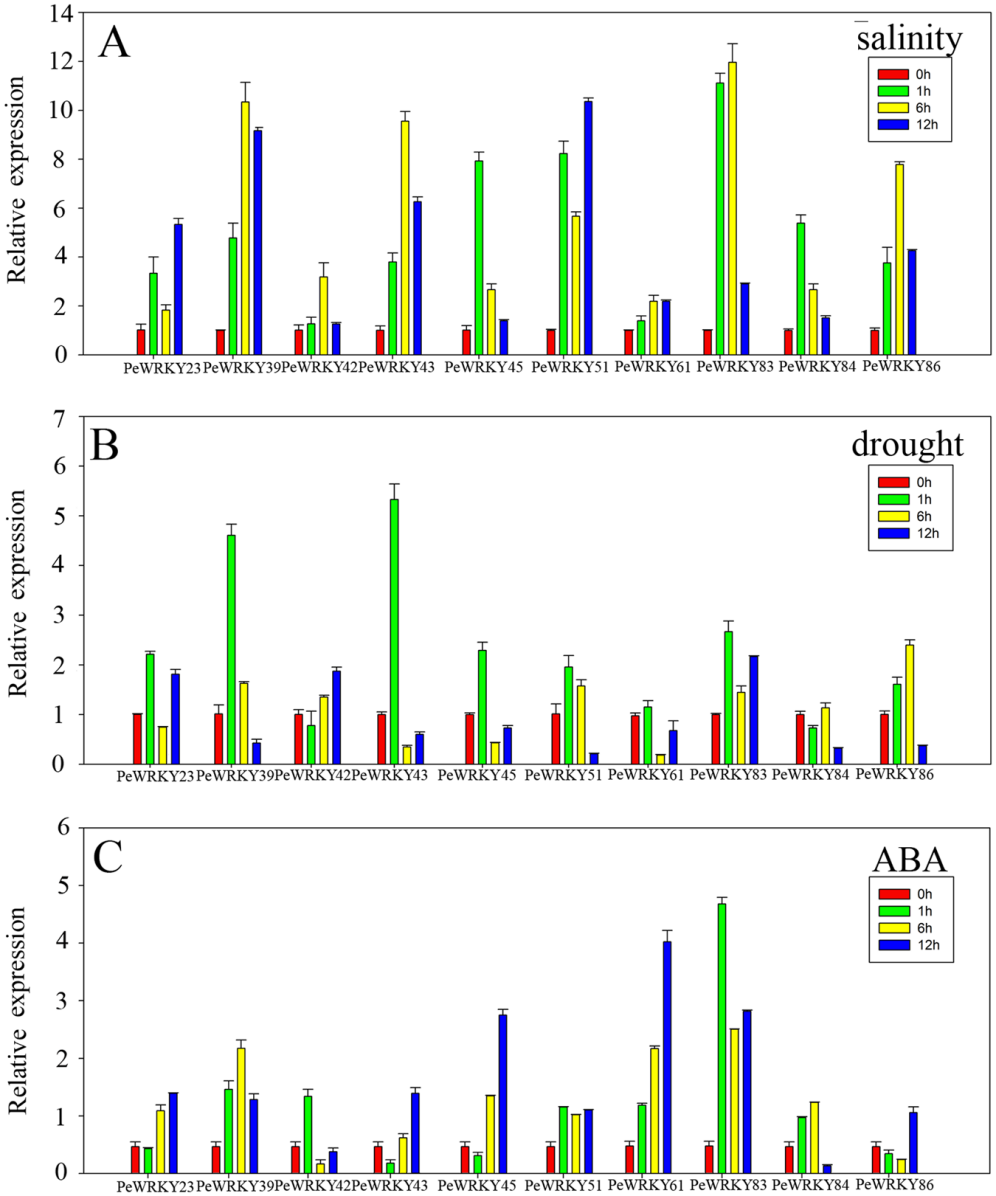
Expression profiles of the *WRKY* IIc genes in 3-month-old moso bamboo seedlings subjected to salinity (**A**), dehydration (**B**) and ABA (**C**) stress treatments. The transcript levels of each *PeWRKY* in the stress-treated plants were plotted as the relative expression (fold) of the non-stressed control plants for 1, 6, and 12 h. The transcript level of Tonoplast intrinsic protein 41 gene (TIP41) was used as a reference. Mean values and standard errors (bar) were shown from three independent experiments.

Expression of the 10 *PeWRKY* genes increased during the first 1 h of salt treatment. The expression of *PeWRKY45*, *-51* and *-83* were increased more than 8-fold (Fig. 4A). Two genes (*PeWRKY42*/84) were down-regulated rapidly following drought treatment. While the expression of the other eight *PeWRKY* genes peaked after 1 h. The expression of *PeWRKY39* was higher 6-fold than the control (Fig. 4B). The expression of most *PeWRKY* genes changed significantly following ABA treatment, except for *PeWRKY84* and *PeWRKY86* (Fig. 4C). For example, *PeWRKY61* was up-regulated more than 10-fold at 12 h. These results further support the hypothesis that *WRKY* genes can play important roles in regulating the response to abiotic stresses.

From the qRT-PCR experimental results, *PeWRKY83* was highly up-regulated following the three treatments and was therefore selected for further investigation (Fig. 4). The full-length cDNA of *PeWRKY83* was comprised of 1074 bp and the deduced protein contained 357 amino acid residues with a predicted molecular mass of 37578.07 Da (Table S1).

### Subcellular localization and DNA-binding assays

**Figure 5:**
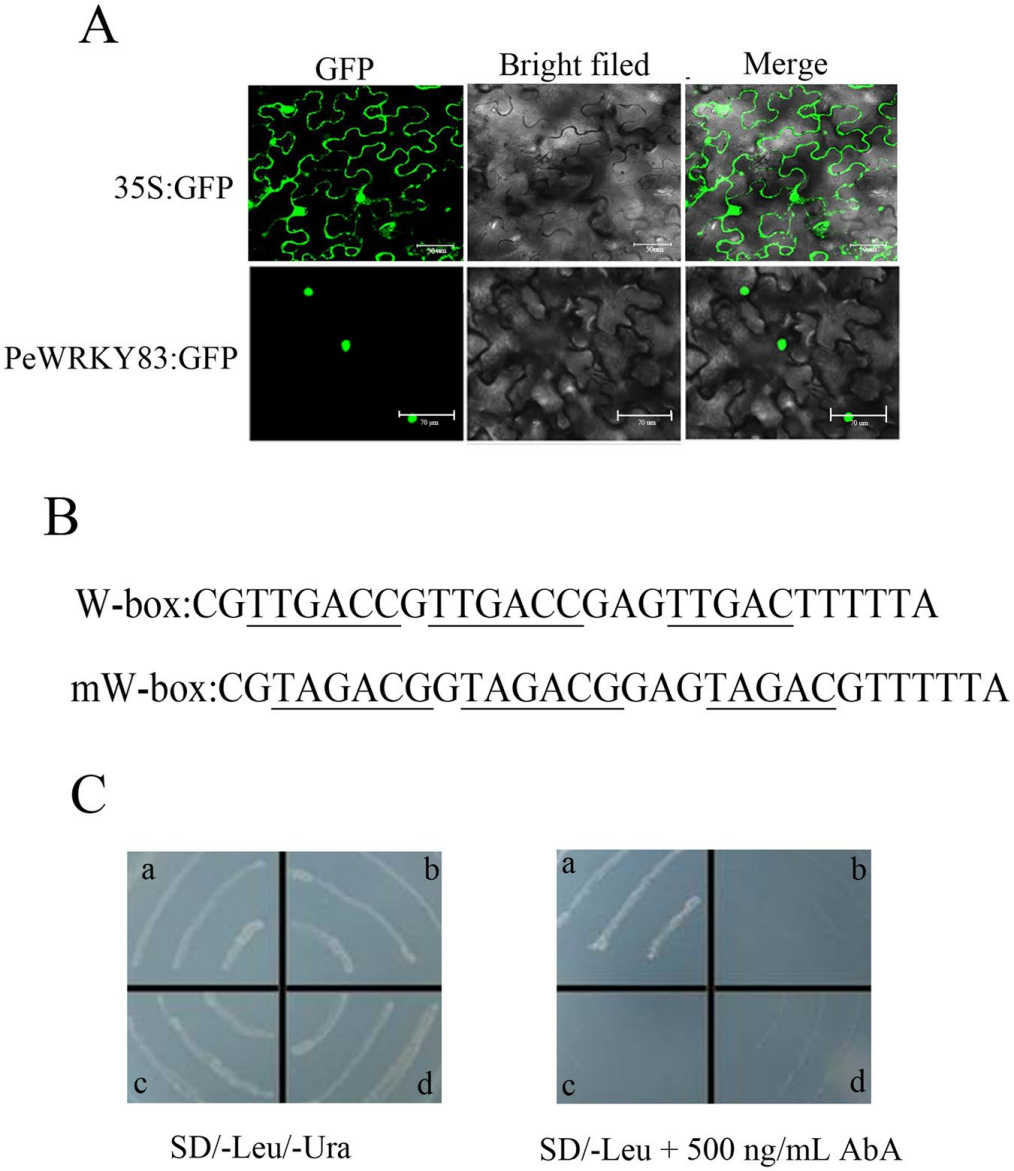
Subcellular localization and DNA-binding assays. (**A**) Subcellular localization of *PeWRKY83*. (**B**) Sequence of the triple tandem repeats of the W-box and mW-box. (**C**) Yeast one-hybrid assay using the 3 × W-box or mW-box as bait. Yeast cells carrying pGAD-*PeWRKY83* or pGAD7 were grown on SD/-Leu/-Ura or SD/-Leu containing 500 ng/ml AbA. (a) pAbAi-W-box/pGAD-*PeWRKY83*; (b) pAbAi-W-box/pGAD7; (c) pAbAi-mW-box/pGAD-*PeWRKY83*; and (d) pAbAi-mW-box/pGAD7.

*PeWRKY83* was located in the nucleus by TargetP 1.1 (http://www.cbs.dtu.dk/services/TargetP/) server and WoLF PSORT (http://wolfpsort.org/). To verify this, p*PeWRKY83*-GFP gene expression vectors were constructed and transformed into *N. tabacum*. As depicted in Fig. 5A, *PeWRKY83* was localized to the nucleus.

Many studies have demonstrated that *WRKY* TFs can bind to the W-box [TTGAC(C/T)] to modulate protein expression. A yeast one-hybrid experiment was used to test whether *PeWRKYs* also have this binding characteristic. First, the two nucleotide chains (W-box/mW-box) were combined manually (Fig. 5B). Subsequently, they were inserted into vector pAbAi and fused to the yeast cells (Y1HGold), forming two strains: pAbAi-W-box and pAbAi-mW-box. Only pGAD-*PeWRKY83*/pAbAiW-box grew on SD/-Leu containing 500 ng/mL AbA (Fig. 5C).

### Protein interaction analysis of *PeWRKY83* with *PeVQs*

**Figure 6:**
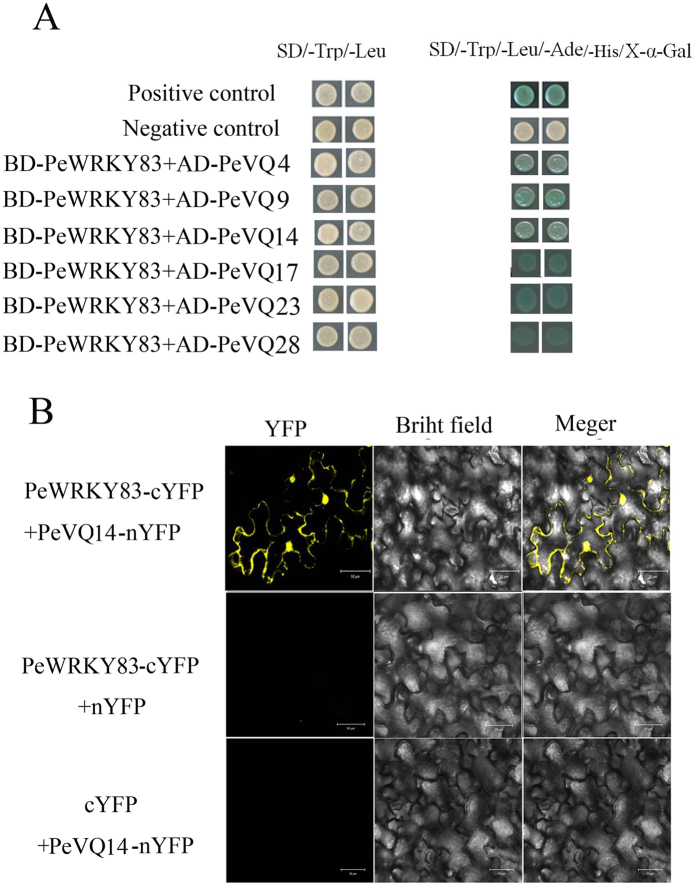
Interaction of *PeWRKY83* with *PeVQs*. (**A**) Interaction of *PeWRKY83* with *PeVQs* in yeast. The bait construct (pGBKT7-*PeWRKY83*) and the prey constructs (pGADT7-*PeVQs*) were co-transformed yeast strain AH109, then examined on SD/-Trp/-Leu and SD/-Trp/-Leu/-Ade/-His/X-α-Gal plates. Positive control, pGBKT7-53 + pGADT7-T; Negative control, pGBKT-53 + pGADT7-Lam. (**B**) BiFC assays of *PeWRKY83* interaction with *PeVQ14 in vivo*. Yellow fluorescent protein (YFP) images were detected at an approximate frequency of 8.46% (1100 of 1300 tobacco leaf epidermal cells analysed exhibited BiFC events). Bars = 50 mm.

In general, the transcription factor cannot work without interaction with other proteins. Herein, *PeWRKY83* showed no transactivation activity in yeast (Figure S3). *WRKY* proteins interact with other proteins, therefore we examined whether *PeWRKY83* would interact with *PeVQ* proteins using a yeast two-hybrid assay. The *PeWRKY83* was fused into pBD, while *PeVQs* were fused into pAD. The fused pBD and pAD vectors were then co-transformed into AH109 yeast cells and grown on SD/-Trp/-Leu/-Ade/-His/X-α-Gal plates for 3–5 days. The interaction ability was tested through ᾳ-galactosidase activity. As shown by the yeast two-hybrid assay (Fig. 6A), *PeWRKY83* interacted with *PeVQs*. *PeVQ14* is localized in the nucleus (data not shown), the same as *PeWRKY83* (Fig. 5A). Figure 6B indicated that *PeWRKY83* interacted with *PeVQ14 in vivo* by BiFC (bimolecular fluorescence complementation) assays in the leaves of tobacco, and the YFP signal was detected in the nuclear compartment of transformed cells.

### Salt tolerance of *PeWRKY83* overexpressing Arabidopsis plants

**Figure 7:**
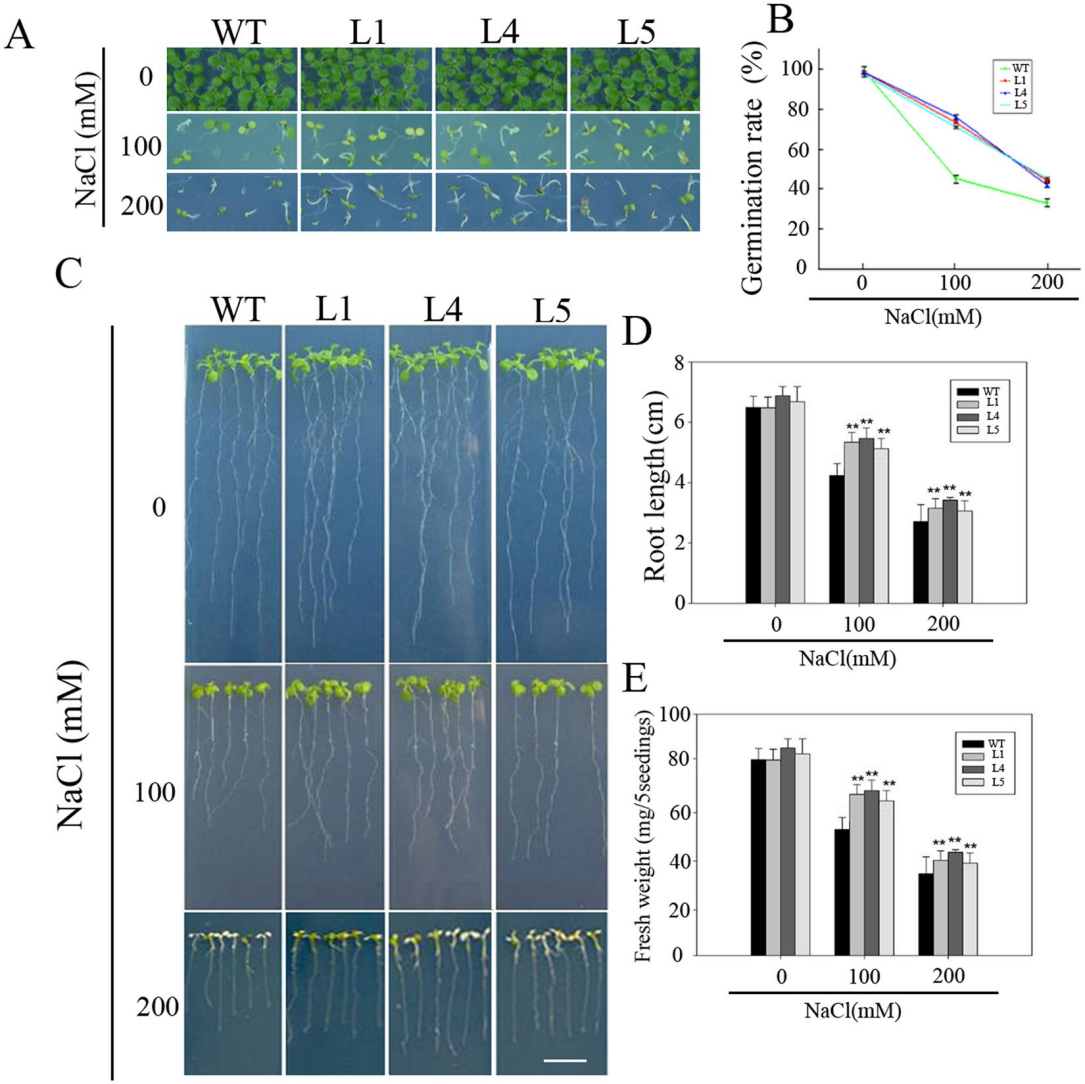
Germination and phenotypes of *PeWRKY83* in transgenic Arabidopsis under salt tolerance. (**A**) Germination performance of *PeWRKY83*-overexpression and WT seeds on 1/2 MS medium containing 0, 100, or 200 mM NaCl measured at 5 d after initiation. (**B**) Calculation of the germination rates of transgenic and WT seeds. (**C** and **D**) Effect of salt stress on root length and fresh weight of transgenic and WT plants. Values are means ± SE (n = 10). *P < 0.05, t-test; **P < 0.01, t-test.

*PeWRKY83*, driven by CaMV 35 S promoter, was transformed into Arabidopsis plants. Three homozygous lines (L1, L4 and L5) with relatively high expression of the transgenosis were further analysed (Figure S4). We first carried out a seed germination experiment on MS plates, containing different concentrations of NaCl. No difference in germination was observed when the three transgenic lines and WT control seeds were grown on 0 mM NaCl MS plates. However, under high salt stress conditions caused by NaCl treatment, the germination rates of both WT and transgenic seeds were inhibited significantly, but the germination rate of WT seeds was significantly lower than that of transgenic seeds (Fig. 7A,B). Similarly, in the presence of 100 or 200 mM NaCl, the transgenic plants showed less growth inhibition than WT (Fig. 7C). The root length of overexpression lines (5.53–5.96 cm) showed significantly lesser suppression than WT (4.34 cm) and the fresh weight also lesser suppression (67.8%–72.57%) than WT (57.31%) under 100 mM NaCl treatment. Likewise, under 200 mM NaCl treatment, the root length of overexpression lines (3.03–3.29 cm) showed significantly lesser suppression than WT (2.75 cm) and the fresh weight also lesser suppression (39.83%–42.36%) than WT (36.43%) (Fig. 7D,E).

**Figure 8:**
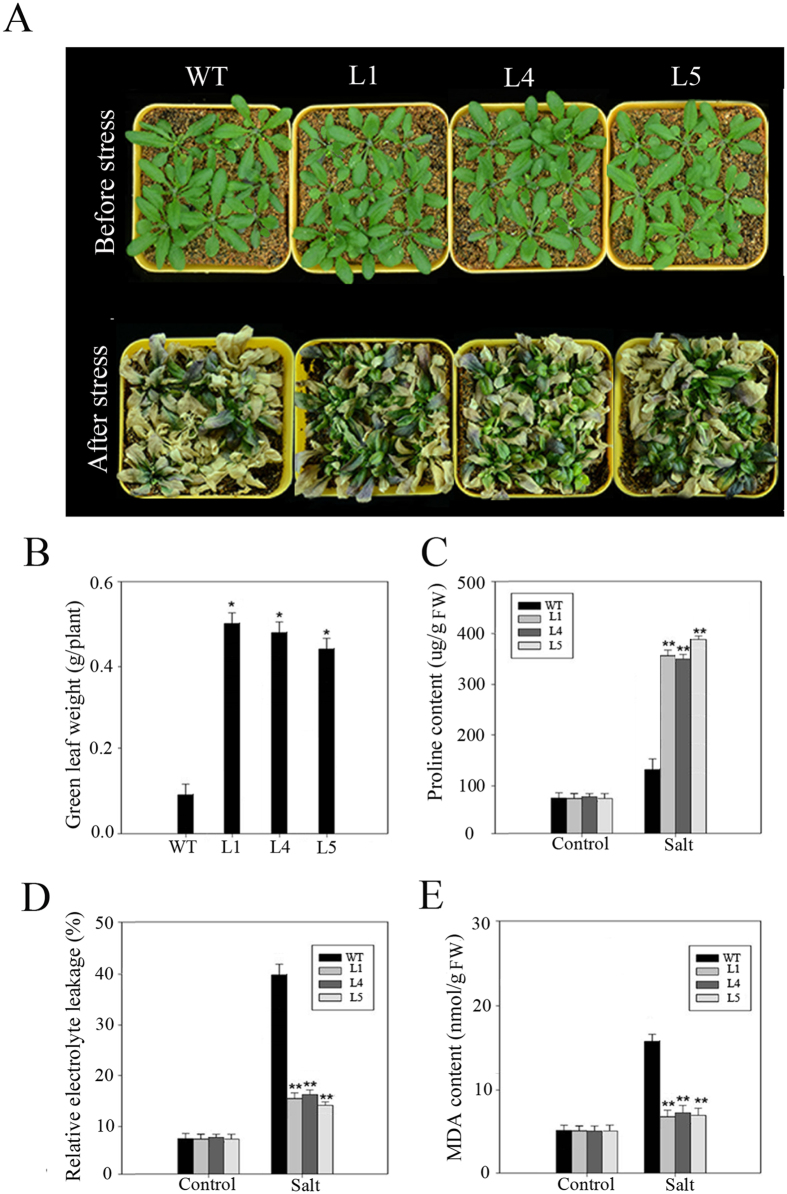
Salt stress of *PeWRKY83* in transgenic Arabidopsis plants. (**A**) Performance of WT and transgenic plants before and after salt treatment with 200 mM NaCl for 20d. (**B**–**E**) Green leaf weight of each plant, proline content, REL and MDA content were measured in WT and transgenic plants after salt treatment. Values are means ± SE (n = 3). *P < 0.05, t-test; **P < 0.01, t-test.

The salt tolerance of transgenic plants grown in soil was also tested. After treatment with 200 mM NaCl for 20 d, most of the transgenic plants grew well, while most of WT plants wilted (Fig. 8A). Green leaf weight of the transgenic plants (0.452–0.506 g/plant) was more weighted than that of the WT plants (0.976 g/plant) (Fig. 8B). The physiological indices of WT and transgenic plants under salt stress were determined. After treatment with 200 mM NaCl, the levels of proline in the transgenic plants was higher (2.3-fold) than in the WT plants (Fig. 8C). As showed in Fig. 8D,E, REL (relative electrolyte leakage) and MDA was not significantly difference between the transgenic plants and WT before stress, yet was significantly lower than the WT plants following salt stress.

### The transgenic Arabidopsis of *PeWRKY83* was less sensitive to ABA

**Figure 9:**
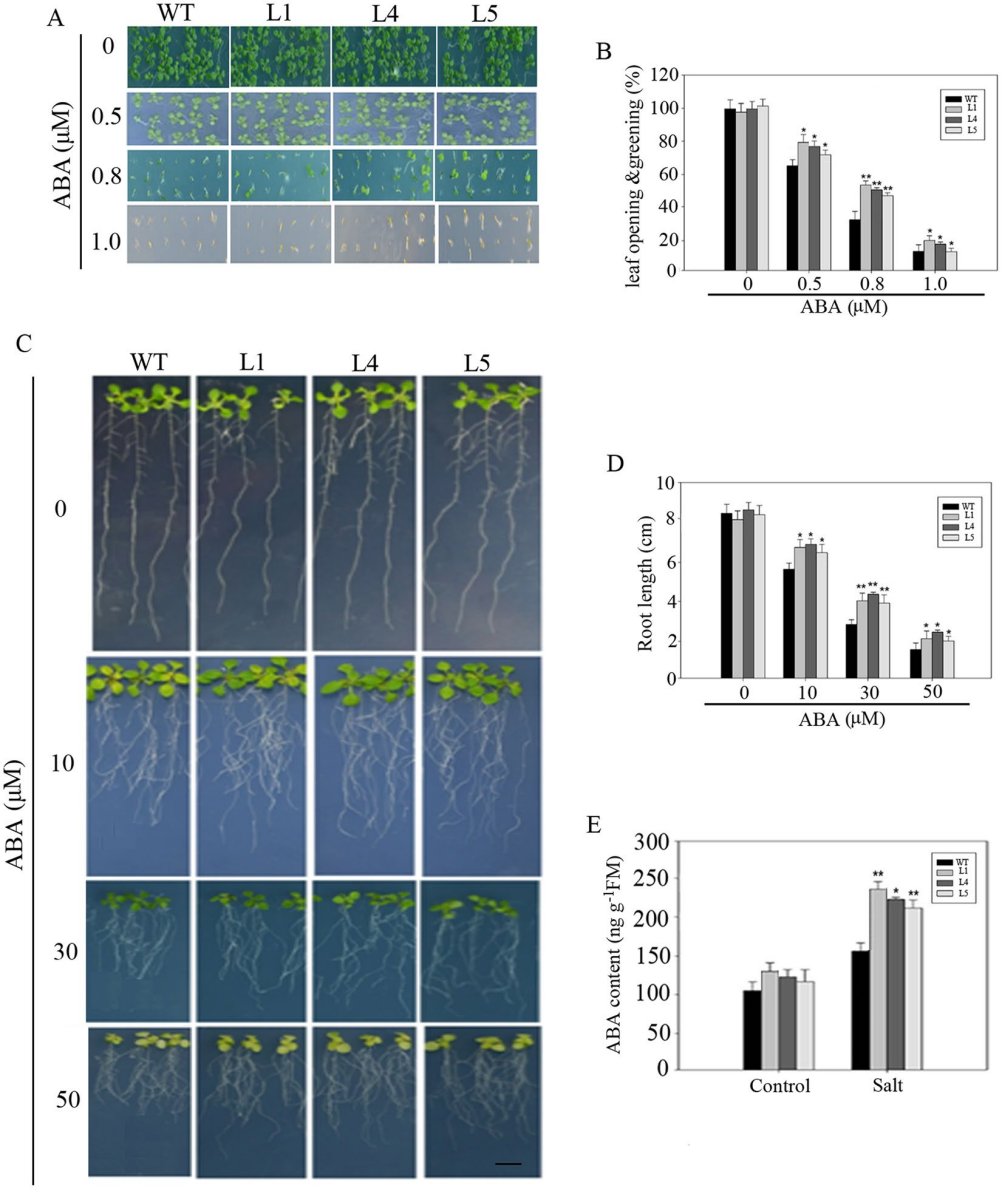
Reduced sensitivity to ABA of *PeWRKY83* in transgenic Arabidopsis. (**A**) Seed germination of transgenic and WT plants on 1/2 MS medium containing different ABA concentrations. (**B**) Statistical analysis of the leaf opening and greening rate in A. (**C** and **D**) Analysis of root length in WT and transgenic Arabidopsis under ABA stress. (**E**) ABA contents in normal or salt treated seedlings. Values are means ± SE (n = 10). *P < 0.05, t-test; **P < 0.01, t-test.

Since *PeWRKY83* was strongly induced by ABA, we further tested if *PeWRKY83* was involved in ABA sensitivity, an important aspect of the ABA-dependent regulation pathway. We tested ABA sensitivity of *PeWRKY83* overexpressing plants at germination and seedling stage. As shown in Fig. 9A, in the normal medium, the germination rate was not significantly different from the WT and transgenic seeds. While in the ABA-supplemented medium, the germination rate of WT seeds was reduced significantly compared with the transgenic seeds. For example, under 0.8 μM ABA treatment, the leaf opening and greening rate of transgenic seeds was 56.76%–58.55%, while the leaf opening and greening rate of the WT seeds was 36% (Fig. 9B). Under different concentrations of ABA treatments, the WT plants were inhibited more severely than the *PeWRKY83* overexpressing seedlings (Fig. 9C). In the medium containing 30 μM ABA, the root length of the transgenic plants (4.23–4.67 cm) was significantly greater than that of the WT plants (3.24 cm) (Fig. 9D). Figure 9E displayed that transgenic and WT plants showed non-significant differences for endogenous ABA content under normal growth conditions, however, it was significantly higher in transgenic seedlings (239–258 ng/g) than the WT (158 ng/g) under salt stress. These results suggest that overexpression of *PeWRKY83* in plants can reduce sensitivity to ABA.

### Expression of ABA-related genes in *PeWRKY83* transgenic Arabidopsis

**Figure 10:**
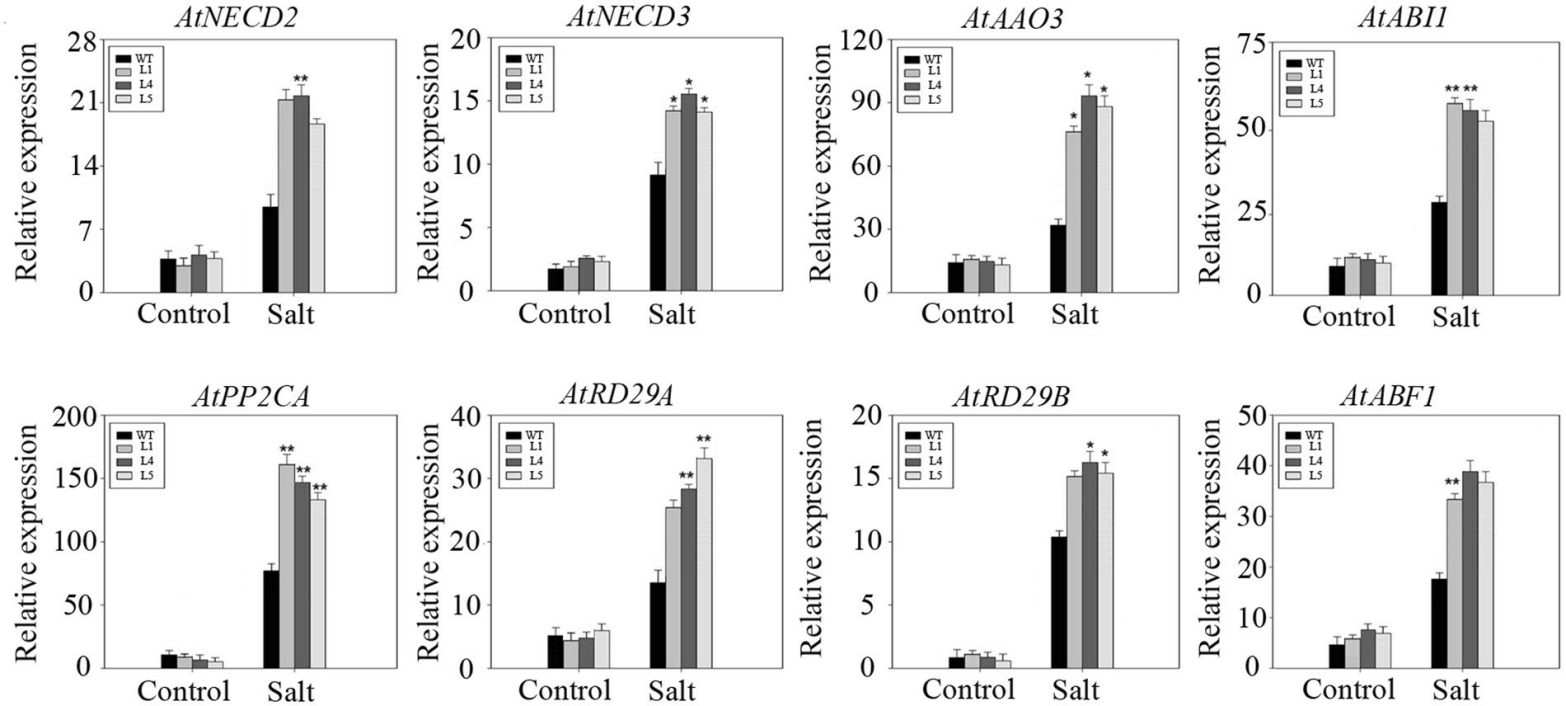
Expression levels of ABA-related genes in *PeWRKY83* transgenic plants. Leaves of WT and transgenic Arabidopsis plants were sampled at 5 d after salt stress. Values are means ± SE (n = 3). *P < 0.05, t-test; **P < 0.01, t-test.

Since the transgenic plants were sensitive to exogenous ABA and accumulated more endogenous ABA under salt stress conditions, the transcription levels of some ABA biosynthesis and signaling genes were analyzed. As shown in Fig. 10, all the genes were strongly induced in the transgenic plants and wild type with salt treatment for 5 d. The transcript levels of three ABA biosynthesis genes (*AtAAO3*, *AtNCED2* and *AtNCED3*) in both the transgenic plants and WT were clearly upregulated by salt stress, while the expression levels of these genes was significantly higher in transgenic plants as compared to WT. The two ABA signalling genes, *AtABI1* and *AtPP2CA*, showed much higher expression levels in transgenic plants than that in WT under salt stress conditions. The expression levels of three well-documented ABA responsive genes, *AtRD29A*, *AtRD29B* and *AtABF1*, showed non-significant differences between the transgenic plants and WT under normal growth conditions. However, under salt stress conditions significant differences were observed for higher expression levels of these genes in transgenic plants as compared to WT were detected. These results suggested that *PeWRKY83* can regulate the expression of ABA-related genes in transgenic Arabidopsis under salt treatment.

## Discussion

Moso bamboo is a fastest-growing plant and most important non-timber forest product in the world. *WRKY* proteins are members of a transcription factor family in higher plants and have a crucial role in plant growth and development. In this study, we identified 89 candidate *WRKY* proteins in *Phyllostachys edulis*. By multiple sequence alignment of their *WRKY* domains, the *PeWRKY* genes were classified into three groups (groups I, II, III). As illustrated in Fig. 1, the *WRKY*GQK signature was highly conserved among moso bamboo *WRKY* proteins, but a slight variation was identified in 9 genes (*PeWRKY7*, *-21*, *-39*, *-43*, *-45*, -51, *-55*, *-61* and *-84*). Especially genes in group IIc had more variation (33.3%) than genes in other *WRKY* groups, which suggested that *WRKY* genes in group IIc are more active and variable. This phenomenon has also been reported in *Arabidopsis thaliana*, *Oryza sativa*, *Hordeum vulgare*, tobacco, canola, sunflower and soybean^6,11,16,30–33^. Based on sequence comparison and phylogenetic analysis, the *PeWRKY* genes were classified into three groups (groups I, II, III), and group II genes were further classified into IIa, IIb, IIc, IId and IIe. The phylogenetic analysis (Fig. 2) showed genes in group IIa clustered with group IIb and that group IId genes are closely related to IIe, which support the classifications of the three subgroups in group II: group IIa/group IIb, group IIc, and group IId/group IIe^16,34^. The Ka/Ks ratios of all *WRKY* paralogous and orthologous pairs were less than 1, suggesting purifying selection (Fig. 3).

The *WRKY* gene family play key roles in plant development and response to various abiotic stresses and, there is increasing evidence which suggest *WRKY* IIc proteins regulate response to abiotic stress. For example, *Arabidopsis WRKY8* was highly up-regulated by salt treatment^35^. Transgenic Arabidopsis overexpressing *TaWRKY19* displayed improved drought and salt stress^36^. A cotton *WRKY* IIc gene, *GhWRKY68*, responded to drought and salt stress^23^. In soybean, there were also two IIc genes (*GmWRKY21*/54) that conferred differential tolerance to abiotic stresses in transgenic Arabidopsis plants^22^. Hence, we randomly selected ten genes from group IIc to study the function of *PeWRKY* IIc genes. Expression profile analysis revealed that *PeWRKY* IIc genes were induced significantly by salt, drought and ABA stresses (Fig. 4). *PeWRKY83* was highly expressed under the three treatments; therefore *PeWRKY83* was selected for investigation.

To study the functions of *PeWRKY83* in plants, we transformed *PeWRKY83* into Arabidopsis. In many reports, Arabidopsis plants have been used in transgenic studies for stress-tolerant genes from species which are not easy to transform including wheat, soybean and moso bamboo^37–39^. Overexpression of *PeWRKY83* improved stress tolerance in transgenic Arabidopsis plants based on phenotype and changes in physiological parameters such as proline, MDA and electrolyte leakage after stress treatments (Figs 7 and 8). The content of proline in *PeWRKY83* transgenic plants was significantly higher than in WT plants under stress conditions. The plants retain water effectively, when the content of proline in plants is increased^40^. REL reflects membrane injury after stresses. MDA is a product of lipid peroxidation in biomembranes^41^. REL and MDA accumulation were reported as indicators of damage caused by abiotic stresses^42,43^. The transgenic plants of *PeWRKY83* had lower MDA content than WT plants under salt stress, suggesting overexpression of *PeWRKY83* in plants can lead to improved tolerance to oxidative stress caused by salt stress. In accordance with the lower content of MDA, overexpression of *PeWRKY83* in Arabidopsis also led to a lower amount of REL compared with WT plants, suggesting that the degree of cell membrane damage in transgenic plants caused by abiotic stress was less than in WT plants.

Seed germination, seedling growth and plant development may be inhibited by ABA^44^. ABA accumulation is induced through ABA-dependent signalling pathways under various stresses^45^. Herein, seed germination and seedling root growth of *PeWRKY83* transgenic plants were inhibited by exogenous ABA (Fig. 9). Moreover, the transgenic lines accumulated more ABA compared with the WT plants during salt stress (Fig. 9F). These results indicate that *PeWRKY83* may increase salt tolerance by positively regulating ABA pathways. This was consistent with previous observations in other plant species^22,23,46^.

In previous studies, several experiments have demonstrated that overexpression of TFs regulate expression of stress/ABA-responsive genes and enhanced tolerance to various stresses by overexpression in plants^47,48^. In our study, expression of the eight marker genes was induced significantly in transgenic *Arabidopsis* plants compared with WT plants under salt treatment (Fig. 10), which indicated *PeWRKY83* may affect expression in the upstream region. The expression of 9-*cis* epoxycarotenoid dioxygenase (NCED) and abscisic acid biosynthetic enzyme (AAO) were generally considered to be participated in ABA biosynthesis^49–52^. These three genes showed similar low expression levels in transgenic plants and WT under normal conditions. While under salt stress conditions, the expression levels of *AtNCED2*, *AtNCED3* and *AtAAO3* in transgenic plants was higher than that in the WT plants. Above results suggested that *PeWRKY83* possible role in stress-induced ABA-biosynthesis along with additional factors. Furthermore, the expression levels of ABA signaling genes(*ABI1* and *AtPP2CA*) and ABA-responsive genes(*AtRD29A*, *AtRD29B* and *AtABF1*) was examined in the transgenic plants and WT under normal and salt stress conditions. *AtABI1* and *AtPP2CA* encode a protein phosphatase 2C, which plays a prime role in ABA-mediated signaling network related to stress responses^53^. Under salt stress conditions, *ABI1* and *AtPP2CA* had higher expression levels in the transgenic plants than that in WT. *AtRD29A*, *AtRD29B* and *AtABF1* have been reported to be marker genes in ABA-dependent stress response way^54,55^. We identified upon salt stress, the expression levels of these genes were significantly higher in the transgenic plants than WT. Overall the results suggested that the overexpression of *PeWRKY83* had further induced ABA synthesis in transgenic plants under salt stress conditions, thus, leading to increased expression levels of ABA signaling and -related genes.

Most transcription factors interact via protein-protein and protein-DNA. The *WRKY* transcription factors can participate in protein-protein interactions with other transcriptional proteins. *AtMEKK1* directly interacts with a senescence-related transcription factor (*AtWRKY53*) at the protein level^56^. Furthermore, Sun *et al*. showed that eleven Arabidopsis *WRKY* proteins (Group IId) can bind to a Ca^2+^-binding signalling protein (calmodulin)^56^. In addition, Cheng *et al*. used yeast two-hybrid assays to show that approximately half of the VQ proteins interact specifically with the *WRKY* domains of group Ic and group IIc *WRKY* proteins^57^. Recently, Chi *et al*. identified HDAC and histone proteins, which can interact with *WRKY* proteins^58^. In this study, we first examined the interaction between *PeWRKY83* and *PeVQs* using yeast two-hybrid assay. In early reports, *AtVQ9* interacted with *AtWRKY8*, and *AtVQ1* interacted with *AtWRKY33* ^3,35^. In moso bamboo, there were 29 non-redundant *VQ* genes, named *VQ1* to *VQ29*, according to their Sequence ID (Table S5). We randomly selected six *PeVQ*s (*PeVQ4*, *PeVQ9*, *PeVQ14*, *PeVQ17*, *PeVQ23* and *PeVQ28*) and found that *PeWRKY83* (IIc) can interact with these six *PeVQs* by yeast two-hybrid assays (Fig. 6A); similar to findings of previous reports^57^. We further verified our result using BiFC assays (Fig. 6B), revealing that *PeWRKY83* can interact with *PeVQ14*. By taking advantage of the results obtained from this study and previous observations, we conclude that the *WRKY* gene family in moso bamboo can take part in the interaction of protein and protein.

## Experimental procedures

### The *WRKY* genes in moso bamboo

To identify the non-redundant *WRKY* genes in the moso bamboo genome, we searched the National Centre for Gene Research database (http://www.ncgr.ac.cn/bamboo)^28^, using the Hidden Markov Model (HMM) profile of the *WRKY* domain (PF03106). All redundant sequences were discarded from further analysis based on Cluster W alignment results^59^. To confirm the *WRKY* domain in identified proteins, domain analysis was performed using Interproscan tool (http://www.ebi.ac.uk/Tools/pfa/iprscan5/)^60^. Furthermore, the *WRKY* domain and zinc-finger motif in the protein sequences was analysed by DNAMAN software and modified manually. Moso bamboo gene information, including chromosome locations, ORF lengths, molecular weight (MW), isoelectric point (pI) and the number of amino acids, were obtained from the Bamboo GDB server (http://www.bamboogdb.org). The *WRKY* proteins were analysed using MEME (Multiple Expectation Maximization for Motif Elicitation) program (http://meme.nbcr.net/meme/cgi-bin/meme.cgi)^61,62^. The ScanProsite database was used to annotate the identified protein motifs^63^.

### Selective pressure analyses

A total of 415 *WRKY* protein sequences (85 *Brachypodium distachyon*, 102 *Oryza sativa*, 136 *Zea maize*, 92 *Triticum aestivum*) were obtained from Phytozome v11.1 (http://www.phytozome.net/), and 46 *Hordeum vulgare WRKY* proteins sequences were downloaded from NCBI databases. Phylogenetic trees were constructed based on the bootstrap neighbour-joining (NJ) method and bootstrap analysis (1,000 replicates) by MEGA 6.0 ^64^. The putative orthologous and paralogous pairs were identified using the two popular methods: phylogeny-based and bidirectional best-hit, these criteria were described in previous studies^65,66^. The variations in selective pressures among these six species were evaluated using PAML software^67^.

### Subcellular localization and DNA-binding assays

The coding region of *PeWRKY83* was cloned and then constructed into pCAMBIAI1305 vector (Clontech, Beijing, country-region China), which contains a CaMV 35 S promoter and *GFP* gene, resulting in fusion *PeWRKY83-GFP* gene. The constructed p*PeWRKY83*-GFP vector was transformed into *Agrobacterium tumefaciens* GV3101 using a Gene Pulser Xcell (BIO-RAD, country-region USA). The suspensions were injected into the leaves of *Nicotiana tabacum*. The expression level of *PeWRKY83*-GFP was observed by confocal laser scanning microscopy (CarlZeiss LSM710, Germany).

The DNA-binding assays were tested by yeast one-hybrid assay as described by Jia *et al*.^23^. Briefly, W-box-specific reporter (pAbAi-Wbox) was used as bait. A yeast effectors vector, pGADT7-*PeWRKY83*, and an empty vector, pGADT7, were transformed into the yeast strain Y1H Gold carrying a pAbAiW-box or a pAbAi-mW-box plasmid. All of the transformed yeast cells were placed on SD/-Leu/-Ura medium and the yeast growth was restrained by Aureobasidin A (AbA). The mutant W-box and mW-box (TAGACG) were used as a negative control.

### Yeast two-hybrid and BiFC assays

To confirm the interaction, the Matchmaker GAL4 two-hybrid system (Clontech, Palo Alto, CA) was used. pBD-*PeWRKY*s and pAD-*PeVQs* fusion constructs were generated from the full length of *PeWRKY83* and *PeVQ* cDNAs, respectively. The prey and bait plasmids were transformed to yeast strain AH109. The transformed yeast cells were plated on selective SD/-Trp/-Leu, or SD/-Trp/-Leu/-Ade/-His plates, at 30 °C for 3–5 days to determine the protein-protein interaction.

The BiFC assays were carried out as previously described^68^. In short, the cDNAs of *PeWRKY83* and *PeVQ14* were cloned into pSPYN(C) E-35S. The resulting constructs (*PeWRKY83*-cYFP and PeVQ14-nYFP) were transformed into Agrobacterium strain GV3101 and infiltrated into the 3-week-old leaves of tobacco (*Nicotiana tabacum*) plants, and analysed after infected 48 h. Fluorescence was observed under a confocal laser scanning microscope (Olympus, http://www.olympus-global.com).

### Plant growth conditions and abiotic stress treatments

Abiotic treatment experiments were performed on three-month-old moso bamboo seedlings, which were grown in an artificial growth chamber with a constant photoperiod (16 h light/8 h darkness) and kept at 22 °C. For dehydration, salinity and ABA treatments, the seedlings were poured into 25% PEG-6000, 200 mM NaCl and 10 μM ABA solutions, respectively. One whole plant was sampled as one replicate and in total four replicates was used in each RNA exaction. Each test was repeated at least three times. Samples for RNA extractions were collected at 0, 1, 6 and 12 h after treatment and stored at −80 °C for RNA isolation.

### Salt tolerance of transgenic Arabidopsis plants

The coding sequence of *PeWRKY83* was cloned into the transgenic vector pCAMBIA1301 under control of CaMV 35 S promoter, and then the vector was transferred into *Agrobacterium tumefaciens* strain GV3101 by freeze–thaw method. Positive transgenic Arabidopsis plants were selected on MS plates containing hygromycin (25 mg/L), homozygous T3 or T4 seeds were used.

For germination, the seeds of transgenic and WT were surface-sterilized and sown on 0, 100 and 200 mM NaCl MS plates, and kept at 4 °C for 3 d (for vernalization). The germination rate was determined after 3–5 d under normal conditions. Transgenic and WT seedlings were germinated normally on MS plates using the same method, then moved to MS plates containing 0, 100 and 200 mM NaCl under normal conditions for 7 d, root length and fresh weight were recorded. These seedlings were further transferred to pots under normal conditions. For salt stress, three-week plants were irrigated with 200 mM NaCl solution for 20 d and phenotypic changes were recorded for both treated and control plants.

### ABA sensitivity test of transgenic plants

First, the seeds of transgenic and WT were germinated on 1/2 MS medium plates, or containing 0.5 μM, 0.8 μM and 1 μM ABA to test germination rate. After 3–5 d, the leaf opening and greening was recorded. The geminated Arabidopsis plantlets of the transgenic seed and WT at the same stage were transferred to 1/2 MS medium with different concentrations of ABA (0, 10, 30 and 50 μM). After 7 days in the greenhouse at 22 °C, root length was measured. ABA was extracted as described previously by Olivella *et al*. (1988), and the ABA content was measured using an ELISA kit (Fangcheng, Beijing, China) according to the manufacturer’s instructions.

### RNA isolation, qRT-PCR analysis

Total RNA was extracted according to the manufacturer’s instructions of the RNAprep Pure Plant Kit (Tiangen). First-strand cDNA was synthesised using a PrimeScriptTM RT Reagent Kit (TaKaRa). Gene-specific primers of *PeWRKYs* and ABA-related genes were designed using Primer Express 3.0 (Table S4). Moso bamboo TIP41 (Genbank: gil242384689) or Arabidopsis Tublin was used as internal controls for normalization of the template cDNA. Real-time quantitative RT-PCR (qRT-PCR) was performed on an ABI 7300 Real-Time system (Applied Biosystems). Each reaction was performed in 20 μl (total volume) and consisted of 10 μl SYBR Green Master mix (Applied Biosystems, USA), 1 pmol of each primer, 2 μl cDNA templates and sterile H_2_O. The steps performed during real-time PCR were as follows: step (1) 50 °C, 2 min; step (2) 95 °C, 7 min; step (3) (95 °C, 5 s; 60 °C, 30 s) × 40 cycles. The data from real-time PCR amplification was estimated in terms of comparative fold expression following 2^−∆∆ct^ method and three biological replicates were carried out.

### Measurement of relative electrolyte leakage (REL), proline and malondialdehyde (MDA) contents

Seedlings of transgenic and control plants were cultured in an artificial climate chamber (22 °C) for three weeks. The seedlings were then subjected to salt treatment with 200 mM NaCl solution for 20 d, the levels of relative electrolyte leakage (REL), proline and malondialdehyde (MDA) in WT and transgenic lines without or with salt treatment were measured by the methods described previously^69,70^.

### Statistical analysis

Data analysis was conducted using SPSS v10.0, and significant differences were determined by Student’s t-test at significance levels of P < 0.01 (**) and P < 0.05 (*).

## Electronic supplementary material

Supplementary information
