# Supplementary material for: A moso bamboo WRKY gene PeWRKY83 confers salinity tolerance in transgenic Arabidopsis plants
Source: Sci Rep. 2017 Sep 15;7:11721. doi: 10.1038/s41598-017-10795-z (PMC5601430; doi:10.1038/s41598-017-10795-z)
Supplement: Supplementary file 1 — Supplementary information [file 41598_2017_10795_MOESM1_ESM.doc]

**A moso bamboo *WRKY* gene *PeWRKY83* confers salinity tolerance in transgenic *Arabidopsis* plants**

**Min Wu^1+^, Huanlong Liu^2+^,Guomin Han^1^, Ronghao Cai^1^, Feng Pan^2^, Yan Xiang^1,2^***

**1** National Engineering Laboratory of Crop Stresses Resistance Breeding, Anhui Agricultural University, Hefei 230036, China.

**2** Laboratory of Modern Biotechnology, School of Forestry and Landscape Architecture, Anhui Agricultural University, Hefei 230036, China.

Corresponding Author, Yan Xiang, Email: [xiangyanahau.@sina.com](mailto:xiangyanahau.@sina.com); Phone: 0551-65781786

**Figure S1. Schematic representation of the 20 conserved motifs in *PeWRKY* proteins.** Motifs of the *PeWRKY* proteins were identified using the online MEME program. Different coloured boxes represent different motifs, with their names in central of the boxes. The coloured boxes were ordered manually according to the results of MEME analysis. The length of each box in the figure does not represent the actual motif size.


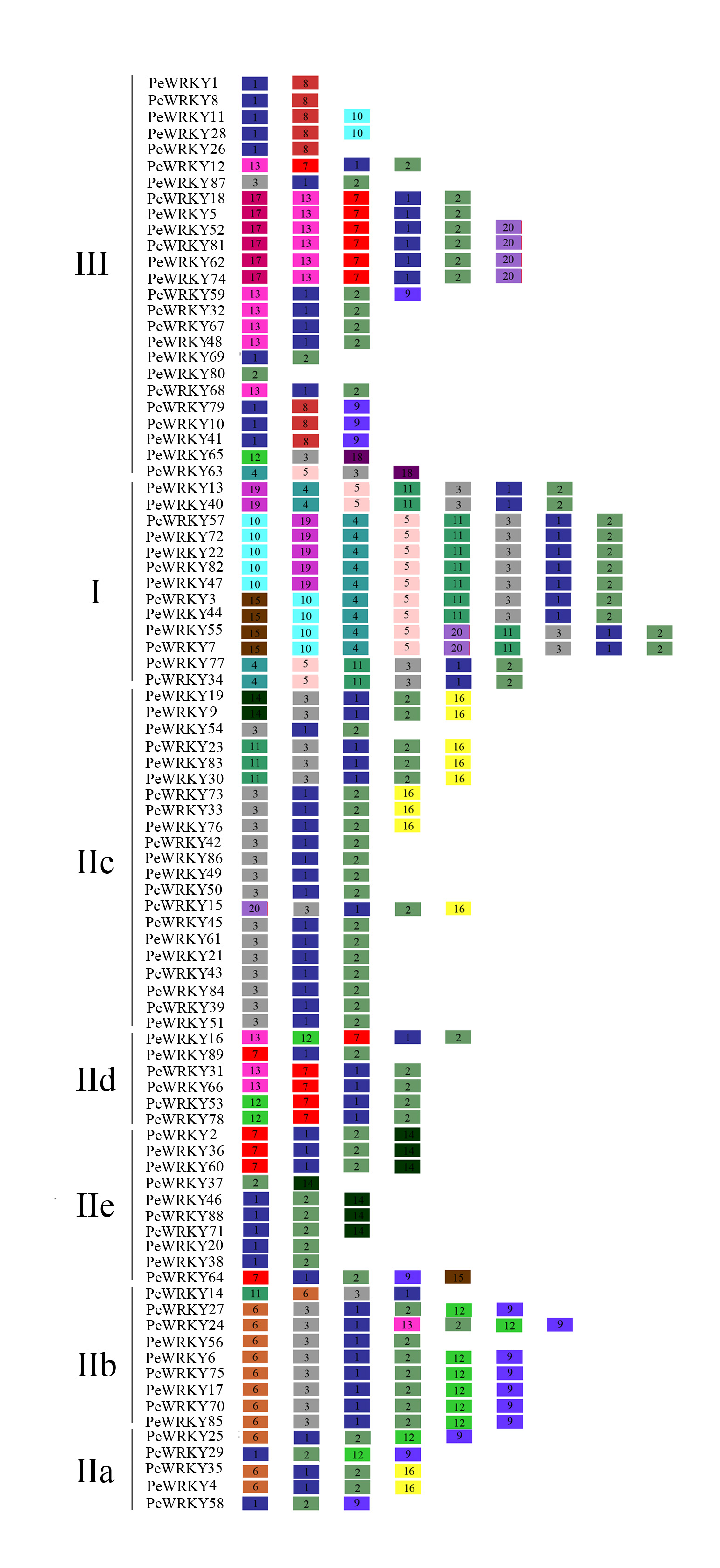


**Figure S2. Phylogenetic tree of *PeWRKYs* constructed by the neighbour-joining method.** Bootstrap values from 1000 replicates are indicated at each node. The branches of different subfamilies are marked by different colours.


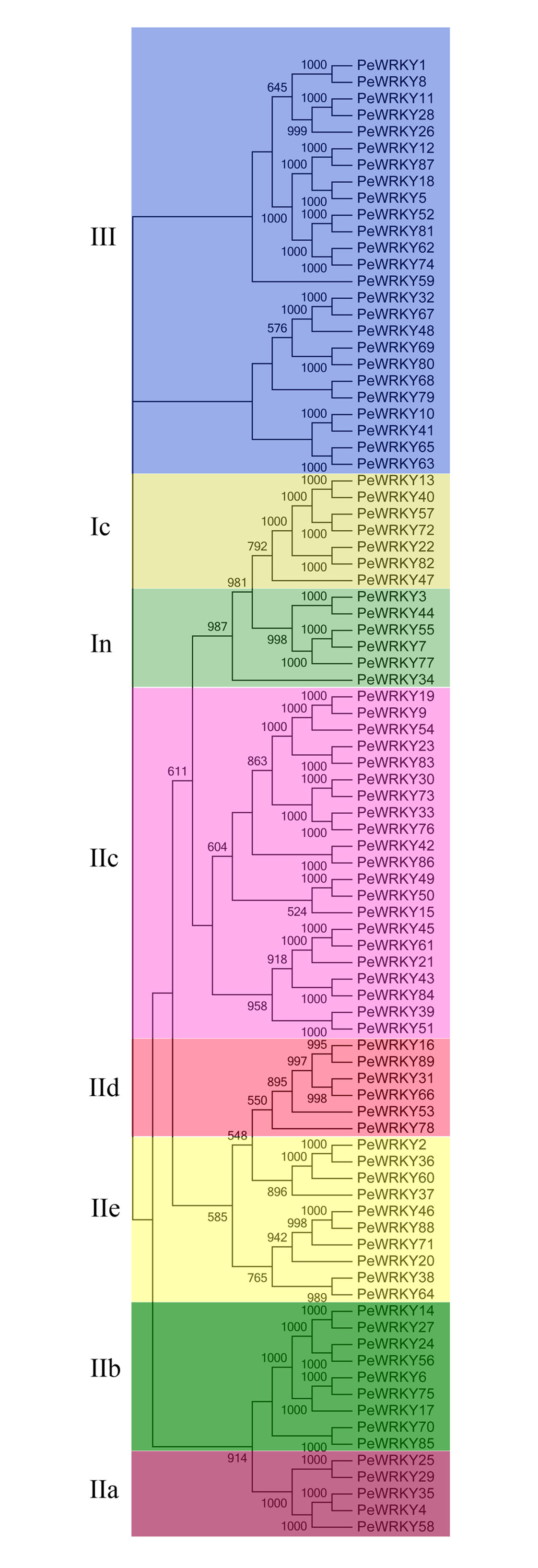


**Figure S3. Transcriptional activation of *PeWRKY83* in yeast.** Positive control, pGBKT7-53+pGADT7-T; Negative control, pGBKT7.

**
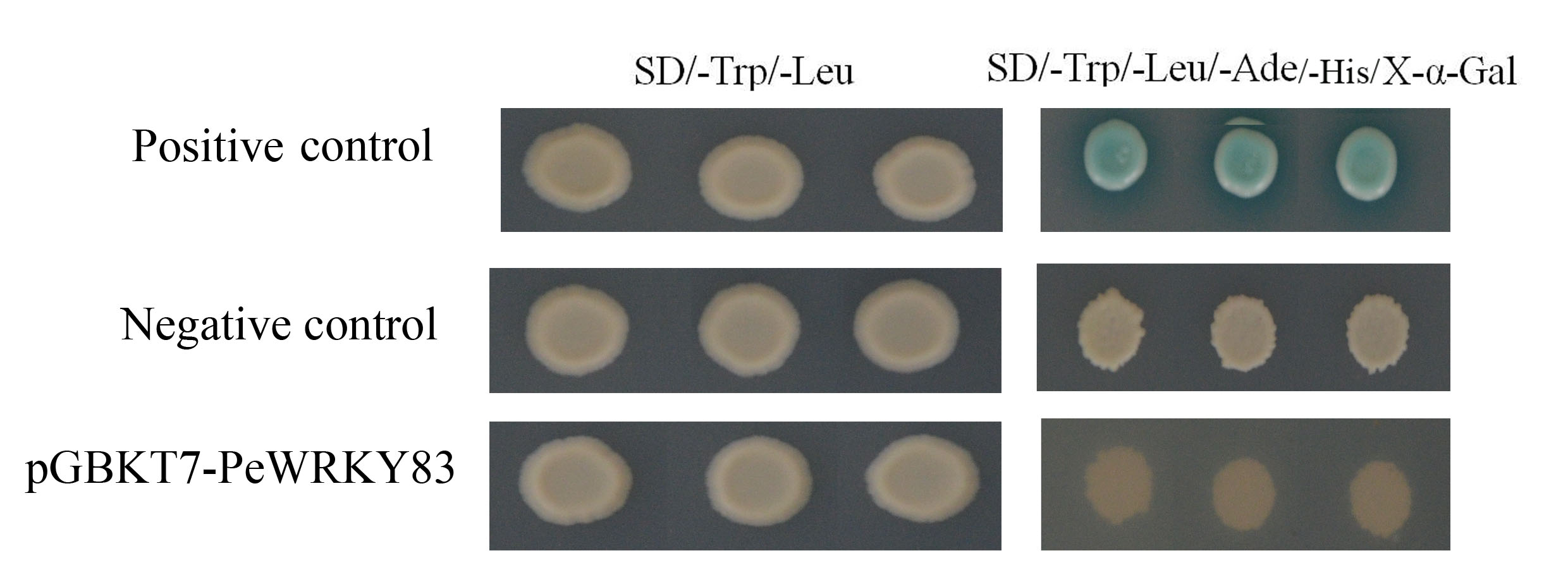
**

**Figure S4. Expression level of *PeWRKY83* in WT and transgenic plants.**


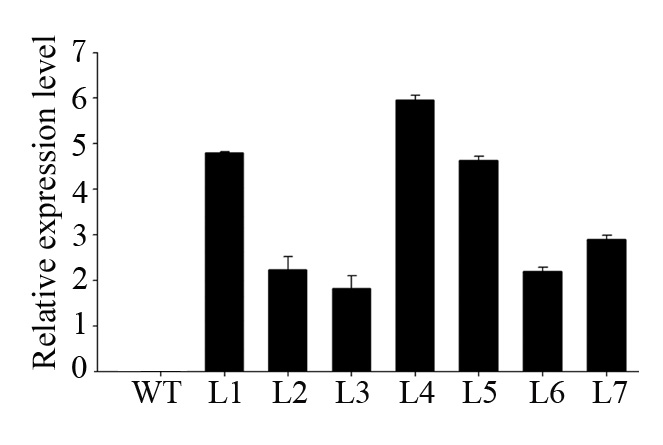


**Table S1. Detailed information on** WRKY proteins in moso bamboo.

| **Gene name** | **Sequence ID** | **Location** |  | | **Size(aa)** | | **Mol.Wt.(Da)** | | **PI** | | **ORF length** |  | | |  | | |
| --- | --- | --- | --- | --- | --- | --- | --- | --- | --- | --- | --- | --- | --- | --- | --- | --- | --- |
| PeWRKY1 | PH01000009G1940 | PH01000009:1253943-1258426(+stand) | | 269 | | 30207.92 | | 6.9 | | 810 | | | |  | | |  |
| PeWRKY2 | PH01000018G0370 | PH01000018:219255 - 220683(- stand) | | 287 | | 31385.08 | | 5.4 | | 864 | | | |  | | |  |
| PeWRKY3 | PH01000035G0560 | PH01000035:412294 - 416795(+ stand) | | 762 | | 81688.87 | | 6.5 | | 2289 | | | |  | | |  |
| PeWRKY4 | PH01000043G1290 | PH01000043:946810 - 948885(+ stand) | | 339 | | 36998.93 | | 8.6 | | 1020 | | |  | | |  | |
| PeWRKY5 | PH01000046G1680 | PH01000046:1229095-1230366(+stand) | | 287 | | 31266.43 | | 6.3 | | 864 | | |  | | |  | |
| PeWRKY6 | PH01000048G1770 | PH01000048:1189533-1194197(+stand) | | 561 | | 58408.46 | | 8.7 | | 1686 | | |  | | |  | |
| PeWRKY7 | PH01000050G1350 | PH01000050:940971 - 945135(+ stand) | | 710 | | 76318.99 | | 6 | | 2133 | | | |  | | |  |
| PeWRKY8 | PH01000112G1110 | PH01000112:842241 - 846946(+ stand) | | 343 | | 38422.92 | | 9 | | 1032 | | |  | | |  | |
| PeWRKY9 | PH01000121G1380 | PH01000121:973352 - 976426(+ stand) | | 339 | | 36900.21 | | 6.6 | | 1020 | | |  | | |  | |
| PeWRKY10 | PH01000130G0430 | PH01000130:266886 - 269295(+ stand) | | 340 | | 36541.63 | | 5.4 | | 1023 | | |  | | |  | |
| PeWRKY11 | PH01000130G0620 | PH01000130:414664 - 421029(+ stand) | | 349 | | 38719.66 | | 9.5 | | 1050 | | |  | | |  | |
| PeWRKY12 | PH01000182G0790 | PH01000182:549439 - 551306(+ stand) | | 309 | | 32442.92 | | 8.5 | | 930 | | | |  | | |  |
| PeWRKY13 | PH01000206G0250 | PH01000206:147206 - 150041(+ stand) | | 560 | | 59323.49 | | 5.8 | | 1683 | | |  | | |  | |
| PeWRKY14 | PH01000212G0380 | PH01000212:295967 - 299691(- stand) | | 291 | | 31398.95 | | 7.1 | | 876 | | |  | | |  | |
| PeWRKY15 | PH01000218G0100 | PH01000218:65611 - 68648(- stand) | | 227 | | 25597.23 | | 8.5 | | 684 | | | |  | | |  |
| PeWRKY16 | PH01000224G1000 | PH01000224:764805 - 766039(- stand) | | 297 | | 31388.11 | | 10 | | 1894 | | | |  | | |  |
| PeWRKY17 | PH01000242G0250 | PH01000242:166837 - 170822(+ stand) | | 561 | | 60004.93 | | 6.4 | | 1686 | | | |  | | |  |
| PeWRKY18 | PH01000242G1360 | PH01000242:872525 - 873698(+ stand) | | 263 | | 29035.81 | | 6.2 | | 792 | | | |  | | |  |
| PeWRKY19 | PH01000256G0030 | PH01000256:32019 - 34153(- stand) | | 340 | | 37291.6 | | 6.3 | | 1023 | | |  | | |  | |
| PeWRKY20 | PH01000256G0230 | PH01000256:170911 - 173019(+ stand) | | 325 | | 35076.2 | | 5.1 | | 978 | | |  | | |  | |
| PeWRKY21 | PH01000271G0420 | PH01000271:313261 - 314626(- stand) | | 219 | | 23129.5 | | 7 | | 660 | | | |  | | |  |
| PeWRKY22 | PH01000296G0390 | PH01000296:250793 - 253057(+ stand) | | 489 | | 51667.96 | | 8.1 | | 1470 | | |  | | |  | |
| PeWRKY23 | PH01000298G0170 | PH01000298:122719 - 125296(- stand) | | 377 | | 40593.39 | | 6.6 | | 1134 | | | |  | | |  |
| PeWRKY24 | PH01000326G0720 | PH01000326:513937 - 522426(- stand) | | 525 | | 54932.19 | | 9.7 | | 1578 | | |  | | |  | |
| PeWRKY25 | PH01000405G0510 | PH01000405:462662 - 464380(+ stand) | | 302 | | 32180.09 | | 8.4 | | 909 | | | |  | | |  |
| PeWRKY26 | PH01000430G0510 | PH01000430:303005 - 304934(+ stand) | | 210 | | 23656.42 | | 8.5 | | 633 | | |  | | |  | |
| PeWRKY27 | PH01000445G0180 | PH01000445:136213 - 141549(+ stand) | | 460 | | 48623.62 | | 6.5 | | 1383 | | |  | | |  | |
| PeWRKY28 | PH01000524G0850 | PH01000524:565086 - 568316(- stand) | | 222 | | 25179.8 | | 9.1 | | 669 | | | |  | | |  |
| PeWRKY29 | PH01000534G0230 | PH01000534:165645 - 168826(+ stand) | | 181 | | 19972.53 | | 6.7 | | 546 | | |  | | |  | |
| PeWRKY30 | PH01000549G0590 | PH01000549:422388 - 426019(+ stand) | | 301 | | 31613.4 | | 6.3 | | 906 | | |  | | |  | |
| PeWRKY31 | PH01000557G0340 | PH01000557:308103 - 309818(+ stand) | | 215 | | 23371.67 | | 9.8 | | 648 | | | |  | | |  |
| PeWRKY32 | PH01000659G0050 | PH01000659:43635 - 44991(+ stand) | | 301 | | 32509.92 | | 4.7 | | 906 | | | |  | | |  |
| PeWRKY33 | PH01000716G0640 | PH01000716:413506 - 417528(- stand) | | 293 | | 31100.66 | | 6.2 | | 882 | | | |  | | |  |
| PeWRKY34 | PH01000716G0820 | PH01000716:532132 - 534846(+ stand) | | 412 | | 44253.05 | | 6.5 | | 1239 | | |  | | |  | |
| PeWRKY35 | PH01000735G0110 | PH01000735:52650 - 54476(+ stand) | | 337 | | 36543.31 | | 6.5 | | 1041 | | |  | | |  | |
| PeWRKY36 | PH01000746G0560 | PH01000746:391694 - 393229(- stand) | | 289 | | 31537.14 | | 5.4 | | 870 | | |  | | |  | |
| PeWRKY37 | PH01000814G0820 | PH01000814:489176 - 491714(- stand) | | 333 | | 36237.62 | | 8.5 | | 1002 | | | |  | | |  |
| PeWRKY38 | PH01000823G0340 | PH01000823:211944 - 213841(- stand) | | 204 | | 22210.98 | | 6.5 | | 615 | | | |  | | |  |
| PeWRKY39 | PH01001004G0240 | PH01001004:205728 - 207391(+ stand | | 242 | | 25618.45 | | 6.3 | | 729 | | | |  | | |  |
| PeWRKY40 | PH01001037G0370 | PH01001037:200393 - 203298(+ stand) | | 505 | | 54108.62 | | 6.1 | | 1518 | | |  | | |  | |
| PeWRKY41 | PH01001070G0190 | PH01001070:168330 - 170874(+ stand) | | 274 | | 29427.66 | | 5.9 | | 825 | | | |  | | |  |
| PeWRKY42 | PH01001100G0250 | PH01001100:210357 - 213609(+ stand) | | 209 | | 22085.99 | | 9.1 | | 630 | | | |  | | |  |
| PeWRKY43 | PH01001131G0560 | PH01001131:365221 - 366633(- stand) | | 189 | | 20660.2 | | 6.6 | | 570 | | | |  | | |  |
| PeWRKY44 | PH01001142G0610 | PH01001142:403867 - 411456(+ stand) | | 568 | | 61359.11 | | 5.5 | | 1707 | | | |  | | |  |
| PeWRKY45 | PH01001210G0270 | PH01001210:138369 - 139991(+ stand) | | 200 | | 21526.86 | | 8.5 | | 603 | | | |  | | |  |
| PeWRKY46 | PH01001232G0560 | PH01001232:393301 - 395604(- stand) | | 358 | | 38384.03 | | 5.3 | | 1077 | | | |  | | |  |
| PeWRKY47 | PH01001245G0180 | PH01001245:107533 - 114338(+ stand) | | 591 | | 63227.44 | | 5.9 | | 1776 | | | |  | | |  |
| PeWRKY48 | PH01001280G0060 | PH01001280:23094 - 24491(+ stand) | | 272 | | 29682.88 | | 8 | | 819 | | | |  | | |  |
| PeWRKY4**9** | PH01001331G0040 | PH01001331:26163 - 27246(+ stand) | | 261 | | 28393.31 | | 9.8 | | 786 | | | |  | | |  |
| PeWRKY50 | PH01001331G0070 | PH01001331:34318 - 35489(+ stand) | | 246 | | 25612.6 | | 8.4 | | 741 | | | |  | | |  |
| PeWRKY51 | PH01001362G0060 | PH01001362:63917 - 65330(+ stand) | | 240 | | 26175.22 | | 8.6 | | 723 | | | |  | | |  |
| PeWRKY52 | PH01001397G0220 | PH01001397:217377 - 219443(+ stand) | | 289 | | 31238.14 | | 7.2 | | 870 | | | |  | | |  |
| PeWRKY53 | PH01001515G0160 | PH01001515:143336 - 144893(- stand) | | 262 | | 28156.57 | | 9.7 | | 789 | | | |  | | |  |
| PeWRKY54 | PH01001601G0220 | PH01001601:126012 - 136038(- stand) | | 333 | | 35825.72 | | 5.7 | | 1022 | | | |  | | |  |
| PeWRKY55 | PH01001626G0320 | PH01001626:176551 - 181054(- stand) | | 699 | | 75339.66 | | 5.8 | | 2100 | | | |  | | |  |
| PeWRKY56 | PH01001671G0400 | PH01001671:305380 - 311411(+ stand) | | 299 | | 32285.05 | | 6.3 | | 900 | | | |  | | |  |
| PeWRKY57 | PH01001737G0080 | PH01001737:40662 - 45059(+ stand) | | 568 | | 60582.18 | | 6.5 | | 1707 | | | |  | | |  |
| PeWRKY58 | PH01001777G0070 | PH01001777:37973 - 39522(+ stand) | | 272 | | 28887.7 | | 9.4 | | 819 | | | |  | | |  |
| PeWRKY59 | PH01001883G0330 | PH01001883:241049 - 245237(- stand) | | 324 | | 34340.38 | | 5.4 | | 975 | | | |  | | |  |
| PeWRKY60 | PH01001953G0270 | PH01001953:126538 - 128094(+ stand) | | 270 | | 29155.6 | | 4.7 | | 813 | | | |  | | |  |
| PeWRKY61 | PH01002011G0380 | PH01002011:216130 - 217478(+ stand) | | 193 | | 20782.91 | | 6.2 | | 582 | | | |  | | |  |
| PeWRKY62 | PH01002018G0330 | PH01002018:224848 - 226283(- stand) | | 36 | | 33997.18 | | 6.6 | | 951 | | | |  | | |  |
| PeWRKY63 | PH01002022G0200 | PH01002022:158875 - 163367(- stand) | | 357 | | 39525.68 | | 5.1 | | 1074 | | | |  | | |  |
| PeWRKY64 | PH01002035G0390 | PH01002035:221869 - 225830(+ stand) | | 488 | | 51194.13 | | 9.9 | | 1467 | | | |  | | |  |
| PeWRKY65 | PH01002142G0270 | PH01002142:192838 - 194281(+ stand) | | 209 | | 21514.26 | | 6.8 | | 630 | | | |  | | |  |
| PeWRKY66 | PH01002220G0280 | PH01002220:225253 - 228458(+ stand) | | 321 | | 33945.51 | | 9.6 | | 966 | | | |  | | |  |
| PeWRKY67 | PH01002238G0110 | PH01002238:72618 - 74469(+ stand) | | 341 | | 36844.43 | | 5.5 | | 1026 | | | |  | | |  |
| PeWRKY68 | PH01002378G0050 | PH01002378:42374 - 43930(- stand) | | 300 | | 31983.52 | | 5.9 | | 903 | | | |  | | |  |
| PeWRKY69 | PH01002396G0160 | PH01002396:95198 - 97177(- stand) | | 310 | | 34150.61 | | 5.6 | | 933 | | | |  | | |  |
| PeWRKY70 | PH01002537G0070 | PH01002537:25620 - 31037(- stand) | | 604 | | 64022.57 | | 7.2 | | 1815 | | | |  | | |  |
| PeWRKY71 | PH01002558G0060 | PH01002558:28183 - 30034(- stand) | | 244 | | 25936.08 | | 7.1 | | 735 | | | |  | | |  |
| PeWRKY72 | PH01002744G0230 | PH01002744:155541 - 158861(- stand) | | 571 | | 61039.91 | | 6.4 | | 1716 | | | |  | | |  |
| PeWRKY73 | PH01002776G0100 | PH01002776:74187 - 78050(- stand) | | 302 | | 31620.26 | | 5.9 | | 909 | | | |  | | |  |
| PeWRKY74 | PH01002800G0110 | PH01002800:57012 - 59891(- stand) | | 312 | | 33754.62 | | 6.2 | | 939 | | | |  | | |  |
| PeWRKY75 | PH01003108G0020 | PH01003108:11957 - 17662(+ stand) | | 529 | | 54936.17 | | 6.6 | | 1590 | | | |  | | |  |
| PeWRKY76 | PH01003110G0050 | PH01003110:47227 - 50751(- stand) | | 301 | | 31719.35 | | 6.7 | | 906 | | | |  | | |  |
| PeWRKY77 | PH01003485G0070 | PH01003485:42286 - 48363(+ stand) | | 921 | | 100478.64 | | 7.7 | | 2766 | | | |  | | |  |
| PeWRKY78 | PH01003558G0150 | PH01003558:134879 - 137020(+ stand) | | 106 | | 11040.49 | | 5.5 | | 321 | | | |  | | |  |
| PeWRKY79 | PH01003579G0140 | PH01003579:114002 - 119009(+ stand) | | 179 | | 20124.54 | | 9 | | 540 | | | |  | | |  |
| PeWRKY80 | PH01003908G0140 | PH01003908:98682 - 101661(+ stand) | | 163 | | 17884.52 | | 4.7 | | 492 | | | |  | | |  |
| PeWRKY81 | PH01003922G0080 | PH01003922:49572 - 52848(- stand) | | 341 | | 36883.41 | | 5.8 | | 1026 | | | |  | | |  |
| PeWRKY82 | PH01004067G0040 | PH01004067:16524 - 19392(+ stand) | | 368 | | 38933.07 | | 8.4 | | 1107 | | | |  | | |  |
| PeWRKY83 | PH01004514G0080 | PH01004514:67730 - 72505(+ stand) | | 357 | | 37578.07 | | 7.8 | | 1074 | | | |  | | |  |
| PeWRKY84 | PH01004940G0100 | PH01004940:58282 - 60192(+ stand) | | 191 | | 20828.1 | | 5.8 | | 576 | | | |  | | |  |
| PeWRKY85 | PH01005137G0020 | PH01005137:7939 - 13772(+ stand) | | 594 | | 62871.38 | | 6.8 | | 1785 | | | |  | | |  |
| PeWRKY86 | PH01005961G0010 | PH01005961:12842 - 16918(- stand) | | 175 | | 20365.62 | | 9.1 | | 528 | | | |  | | |  |
| PeWRKY87 | PH01021666G0010 | PH01021666:1653 - 2443(- stand) | | 223 | | 23685.21 | | 6.3 | | 672 | | | |  | | |  |
| PeWRKY88 | PH01029174G0010 | PH01029174:974 - 2169(- stand) | | 253 | | 26480.4 | | 5.8 | | 762 | | | |  | | |  |
| PeWRKY89 | PH01278028G0010 | PH01278028:27 - 501(- stand) | | 163 | | 10217.76 | | 10.4 | | 492 | | |  | | |  | |

**Table S2. The major MEME motif sequences** **and lengths in *PeWRKY* proteins.**

| **Motif** | **Width** | **Conserved amino acid sequences** |
| --- | --- | --- |
| 1 | 21 | WRKYGQKMIKNNPYPRSYYRC |
| 2 | 29 | GCPVKKQVERCREDPSMVITTYEGKHNHP |
| 3 | 21 | REPRVVFQTRSEIDILDDGYR |
| 4 | 43 | PAEDGYNWRKYGQKQVKNSENPRSYYKCTHPNCPVKKKVERSL |
| 5 | 28 | DGQITEIIYKGTHNHPKPQNNRRNSVPF |
| 6 | 41 | ELAAMEAELRRVNEENRRLRKMLTEVTRSYQALYMHFYQMM |
| 7 | 29 | KKRKNMVKWTIQVPVISAKIADIPADDYH |
| 8 | 37 | TYKNDMKCPATKQVQQKDTEDPPLFRVIYFNEHTCNS |
| 9 | 28 | NLVEQMAMAITRDPNFTTALAAAISSII |
| 10  11  12  13  14  15  16  17  18  19  20 | 22  29  23  19  29  43  19  29  11  21  29 | YFNIPPGLSPATLLESPVFLTN  GCEFDDDEPESKRWKKDGDGEGISMAGNR  CSSTMCTISISAPFPTITLDLTN  CRHLCAQIISCCDRSIGII  PLPEEEEWERELQGEDMLFAGLGELPECN  QMDIQPEPNLPHFNQKSNSHSGLAERMQARAGFSVPKIDMPCI  MPFDYGLLQDMVPPFMRHN  MNYCGGNHQLVSELSHIKELVRQLEVHLH  WRKYGQKVIKC  SIFPSPTTGAFPSQPFNWMPT  PKIHPIHNPHAQCLLQSLSLWLTVKTECL |

**Table S3**. Ka/Ks analysis for the identified paralogous and orthologous among six grass plants.

| **paralogous genes** | **Ka** | **Ks** | **Ka/Ks** |
| --- | --- | --- | --- |
| ZmWRKY102-ZmWRKY41 | 0.3967 | 0.5512 | 0.72 |
| ZmWRKY116.1-ZmWRKY116.2 | 0.10134 | 0.24355 | 0.416 |
| ZmWRKY103-ZmWRKY33 | 0.1007 | 0.20101 | 0.501 |
| ZmWRKY70.1-ZmWRKY70.2 | 0.03546 | 0.22797 | 0.156 |
| ZmWRKY92.1-ZmWRKY92.2 | 0.10134 | 0.24355 | 0.416 |
| ZmWRKY82.1-ZmWRKY82.2 | 0.08358 | 0.22833 | 0.366 |
| ZmWRKY81.1-ZmWRKY81.2 | 0.19539 | 0.33221 | 0.588 |
| ZmWRKY117-ZmWRKY16 | 0.3107 | 0.4501 | 0.69 |
| ZmWRKY31-ZmWRKY65 | 0.5246 | 0.7973 | 0.658 |
| ZmWRKY110.1-ZmWRKY110.2 | 0.2134 | 0.4355 | 0.49 |
| ZmWRKY105-ZmWRKY37 | 0.3582 | 0.8331 | 0.43 |
| ZmWRKY19-ZmWRKY54 | 0.3539 | 0.4221 | 0.838 |
| ZmWRKY75.1-ZmWRKY75.2 | 0.1511 | 0.2703 | 0.559 |
| ZmWRKY27-ZmWRKY86 | 0.1764 | 0.3622 | 0.487 |
| ZmWRKY113-ZmWRKY53 | 0.26299 | 0.7056 | 0.373 |
| ZmWRKY78-ZmWRKY91 | 0.31008 | 0.54782 | 0.566 |
| ZmWRKY49-ZmWRKY5 | 0.05525 | 0.13369 | 0.413 |
| ZmWRKY9.1-ZmWRKY9.2 | 0.10791 | 0.36197 | 0.298 |
| ZmWRKY100-ZmWRKY42 | 0.12239 | 0.19582 | 0.625 |
| ZmWRKY25.1-ZmWRKY25.2 | 0.3466 | 0.5534 | 0.63 |
| ZmWRKY45-ZmWRKY97 | 0.29737 | 0.35895 | 0.828 |
| ZmWRKY13.1-ZmWRKY13.2 | 0.3146 | 0.46089 | 0.683 |
| ZmWRKY26-ZmWRKY85 | 0.11995 | 0.26561 | 0.452 |
| ZmWRKY15.1-ZmWRKY15.2 | 0.2085 | 0.34247 | 0.609 |
| ZmWRKY24-ZmWRKY46 | 0.15367 | 0.40353 | 0.381 |
| ZmWRKY8-ZmWRKY58 | 0.05192 | 0.12997 | 0.399 |
| ZmWRKY6-ZmWRKY60 | 0.0732 | 0.33899 | 0.216 |
| ZmWRKY7-ZmWRKY59 | 0.11458 | 0.15652 | 0.732 |
| ZmWRKY43-ZmWRKY99 | 0.09242 | 0.19833 | 0.66 |
| ZmWRKY4-ZmWRKY30 | 0.0872 | 0.48117 | 0.181 |
| ZmWRKY68.1-ZmWRKY68.2 | 0.15838 | 0.21678 | 0.731 |
| ZmWRKY48-ZmWRKY114 | 0.11394 | 0.28332 | 0.401 |
| ZmWRKY109-ZmWRKY66 | 0.15681 | 0.24529 | 0.639 |
| ZmWRKY79.1-ZmWRKY79.2 | 0.16649 | 0.24621 | 0.676 |
| ZmWRKY118-ZmWRKY14 | 0.06457 | 0.29068 | 0.222 |
| ZmWRKY44-ZmWRKY98 | 0.13612 | 0.22848 | 0.596 |
| ZmWRKY51-ZmWRKY64 | 0.11089 | 0.26575 | 0.417 |
| ZmWRKY57-ZmWRKY62 | 0.07505 | 0.15764 | 0.476 |
| ZmWRKY67-ZmWRKY84 | 0.15692 | 0.34267 | 0.453 |
| OsWRKY89a-OsWRKY89b | 0.0285 | 0.1078 | 0.26 |
| OsWRKY80-OsWRKY90 | 0.5745 | 0.75284 | 0.76 |
| OsWRKY57-OsWRKY87 | 0.21674 | 0.41255 | 0.53 |
| OsWRKY55a-OsWRKY55b | 0.0213 | 0.0246 | 0.87 |
| OsWRKY46a-OsWRKY46b | 0.51358 | 0.78487 | 0.65 |
| OsWRKY97-OsWRKY83 | 0.5745 | 0.78744 | 0.73 |
| OsWRKY40-OsWRKY64 | 0.05333 | 0.0269 | 0.575 |
| OsWRKY50-OsWRKY65 | 0.3151 | 0.3578 | 0.88 |
| OsWRKY75-OsWRKY91 | 0.6041 | 0.8597 | 0.7 |
| OsWRKY61-OsWRKY63 | 0.57941 | 0.97875 | 0.592 |
| OsWRKY56-OsWRKY95 | 0.0978 | 0.1267 | 0.77 |
| OsWRKY38-OsWRKY59 | 0.1808 | 0.4878 | 0.37 |
| BdWRKY59-BdWRKY67 | 0.00528 | 0.01137 | 0.465 |
| BdWRKY47-BdWRKY7 | 0.09455 | 0.33418 | 0.283 |
| BdWRKY9-BdWRKY10 | 0.7537 | 0.8976 | 0.84 |
| BdWRKY17-BdWRKY78 | 0.3037 | 0.4114 | 0.74 |
| BdWRKY15-BdWRKY29 | 0.16325 | 0.34758 | 0.47 |
| BdWRKY24-BdWRKY26 | 0.58251 | 0.9321 | 0.62 |
| BdWRKY49-BdWRKY57 | 0.57167 | 0.85118 | 0.67 |
| HvWRKY19-HvWRKY20 | 0.18075 | 0.24785 | 0.729 |
| HvWRKY1-HvWRKY38 | 0.00161 | 0.20621 | 0.0078 |
| HvWRKY2-HvWRKY23 | 0.04611 | 0.13213 | 0.349 |
| TaWRKY33-TaWRKY35 | 0.05995 | 0.31625 | 0.19 |
| TaWRKY38-TaWRKY83 | 0.27107 | 0.34688 | 0.781 |
| TaWRKY58-TaWRKY87 | 0.1508 | 0.45483 | 0.332 |
| TaWRKY54-TaWRKY86 | 0.16871 | 0.37283 | 0.453 |
| TaWRKY89-TaWRKY91 | 0.01337 | 0.09946 | 0.134 |
| TaWRKY75-TaWRKY88 | 0.01092 | 0.03645 | 0.299 |
| TaWRKY27-TaWRKY71 | 0.18932 | 0.35585 | 0.532 |
| TaWRKY60-TaWRKY61 | 0.20071 | 0.40324 | 0.498 |
| TaWRKY26-TaWRKY34 | 0.13952 | 0.24169 | 0.577 |
| TaWRKY74-TaWRKY81 | 0.02366 | 0.08013 | 0.295 |
| TaWRKY2-TaWRKY80 | 0.01325 | 0.09315 | 0.142 |
| TaWRKY85-TaWRKY92 | 0.36442 | 0.59892 | 0.608 |
| TaWRKY40-TaWRKY65 | 0.49987 | 0.67729 | 0.738 |
| TaWRKY20-TaWRKY46 | 0.44206 | 0.59495 | 0.743 |
| TaWRKY10-TaWRKY7 | 0.01084 | 0.03558 | 0.305 |
| TaWRKY23-TaWRKY8 | 0.23035 | 0.37507 | 0.614 |
| TaWRKY56-TaWRKY22 | 0.05545 | 0.27206 | 0.204 |
| PeWRKY57-PeWRKY72 | 0.04367 | 0.18119 | 0.241 |
| PeWRKY13-PeWRKY40 | 0.0475 | 0.12466 | 0.381 |
| PeWRKY22-PeWRKY82 | 0.09143 | 0.2111 | 0.433 |
| PeWRKY55-PeWRKY7 | 0.04849 | 0.14479 | 0.335 |
| PeWRKY3-PeWRKY44 | 0.07101 | 0.18178 | 0.391 |
| PeWRKY1-PeWRKY8 | 0.09222 | 0.22235 | 0.415 |
| PeWRKY9-PeWRKY19 | 0.03734 | 0.13405 | 0.279 |
| PeWRKY39-PeWRKY51 | 0.12555 | 0.23494 | 0.534 |
| PeWRKY33-PeWRKY76 | 0.0509 | 0.13686 | 0.372 |
| PeWRKY46-PeWRKY88 | 0.4972 | 0.17156 | 0.29 |
| PeWRKY24-PeWRKY56 | 0.10805 | 0.21961 | 0.492 |
| PeWRKY70-PeWRKY85 | 0.06015 | 0.13525 | 0.445 |
| PeWRKY45-PeWRKY61 | 0.04409 | 0.1628 | 0.271 |
| PeWRKY43-PeWRKY84 | 0.07151 | 0.08374 | 0.854 |
| PeWRKY12-PeWRKY87 | 0.83787 | 0.98775 | 0.85 |
| PeWRKY5-PeWRKY18 | 0.11042 | 0.16104 | 0.686 |
| PeWRKY11-PeWRKY28 | 0.10744 | 0.31532 | 0.341 |
| PeWRKY10-PeWRKY41 | 0.13907 | 0.28251 | 0.492 |
| PeWRKY52-PeWRKY81 | 0.5918 | 0.621 | 0.95 |
| PeWRKY4-PeWRKY35 | 0.69241 | 0.7456 | 0.93 |
| PeWRKY30-PeWRKY73 | 0.2958 | 0.53761 | 0.56 |
| PeWRKY6-PeWRKY75 | 0.09037 | 0.17189 | 0.526 |
| PeWRKY14-PeWRKY27 | 0.09264 | 0.22062 | 0.42 |
| PeWRKY69-PeWRKY80 | 0.06582 | 0.06756 | 0.974 |
| PeWRKY32-PeWRKY67 | 0.06788 | 0.17595 | 0.386 |
| PeWRKY49-PeWRKY50 | 0.05918 | 0.20621 | 0.287 |
| **orthologous genes** | **Ka** | **Ks** | **Ka/Ks** |
| BdWRKY38-HvWRKY6 | 0.4381 | 0.9101 | 0.48 |
| BdWRKY28-HvWRKY4 | 0.23639 | 0.67608 | 0.35 |
| BdWRKY48-HvWRKY37 | 0.15618 | 0.41095 | 0.38 |
| BdWRKY74-HvWRKY9 | 0.06161 | 0.44356 | 0.139 |
| PeWRKY34-BdWRKY27 | 0.18781 | 0.54108 | 0.347 |
| HvWRKY32-TaWRKY45 | 0.23668 | 0.29422 | 0.804 |
| HvWRKY21-TaWRKY63 | 0.03684 | 0.14565 | 0.253 |
| HvWRKY25-TaWRKY47 | 0.15437 | 0.29173 | 0.529 |
| HvWRKY10-TaWRKY52 | 0.47863 | 0.97433 | 0.49 |
| HvWRKY15-TaWRKY3 | 0.02404 | 0.08475 | 0.284 |
| BdWRKY20-OsWRKY79 | 0.33934 | 0.56274 | 0.603 |
| BdWRKY12-OsWRKY32 | 0.5215 | 0.6422 | 0.81 |
| BdWRKY81-OsWRKY92 | 0.3 | 0.75016 | 0.4 |
| ZmWRKY11-HvWRKY11 | 0.08174 | 0. 41427 | 0.197 |
| ZmWRKY32-BdWRKY3 | 0.35833 | 0.91993 | 0.39 |
| ZmWRKY12-BdWRKY73 | 0.5894 | 0.9379 | 0.63 |
| HvWRKY16-OSWRKY67 | 0.63313 | 0.7217 | 0.877 |
| PeWRKY65-TaWRKY55 | 0.7783 | 0.9951 | 0.78 |
| OsWRKY20-ZmWRKY104 | 0.5796 | 0.6712 | 0.86 |
| OsWRKY47-ZmWRKY83 | 0.40786 | 0.71495 | 0.57 |
| OsWRKY51-ZmWRKY17 | 0.11651 | 0.49424 | 0.236 |
| OsWRKY2-ZmWRKY3 | 0.40011 | 0.98318 | 0.407 |
| PeWRKY59-ZmWRKY69 | 0.23359 | 0.57859 | 0.404 |
| PeWRKY89-ZmWRKY119 | 0.74778 | 0.76575 | 0.977 |
| PeWRKY23-ZmWRKY74 | 0.21819 | 0.4825 | 0.452 |
| PeWRKY47-OsWRKY78 | 0.06248 | 0.41071 | 0.152 |
| PeWRKY79-OsWRKY60 | 0.33638 | 0.46562 | 0.722 |
| PeWRKY17-OsWRKY43 | 0.19942 | 0.36181 | 0.551 |
| PeWRKY53-OsWRKY42 | 0.3758 | 0.8783 | 0.43 |
| PeWRKY58-OsWRKY28 | 0.3158 | 0.7835 | 0.40 |
| PeWRKY20-OsWRKY12 | 0.17791 | 0.25585 | 0.695 |
| PeWRKY64-OsWRKY66 | 0.1112 | 0.51554 | 0.371 |
| PeWRKY29-OsWRKY62 | 0.16836 | 0.69939 | 0.241 |
| PeWRKY83-OsWRKY16 | 0.18826 | 0.29844 | 0.631 |
| PeWRKY54-OsWRKY8 | 0.2443 | 0.45746 | 0.534 |
| PeWRKY38-OsWRKY41 | 0.10888 | 0.29449 | 0.37 |
| TaWRKY48-ZmWRKY35 | 0.39496 | 0.60963 | 0.648 |
| TaWRKY17-OsWRKY34 | 0.30705 | 0.4173 | 0.736 |
| BdWRKY85-TaWRKY50 | 0.82357 | 0.9543 | 0.86 |
| BdWRKY11-TaWRKY76 | 0.7519 | 0.86 99 | 0.86 |
| BdWRKY5-TaWRKY1 | 0.2 543 | 0.3932 | 0.647 |
| BdWRKY31-TaWRKY5 | 0. 6762 | 0.8267 | 0.82 |
| 0.415BdWRKY69-TaWRKY31 | 0.4571 | 0.6174 | 0.74 |

## Table S4. Primer sequences

|  | **Primers for qRT-PCR analysis** | |  |
| --- | --- | --- | --- |
| **Genes** | **Primers used in qRT-PCR(5′→3′)** | |  |
| *PeWRKY23* | Forwards： | AGAACAGCCCATTTCCAAGGA |  |
|  | Reverse： | GCTTCTTCACCGGGCACTT |  |
| *PeWRKY39* | Forwards： | ACGGGAAGAAGATGGTCAAGAA |  |
|  | Reverse： | CGCTCGAGCAACGGTAGTAGT |  |
| *PeWRKY 42* | Forwards： | AAGAAGCAGGTGCAGAGACTATCC |  |
|  | Reverse： | TGCGTGCCCTCGTACGT |  |
| *PeWRKY43* | Forwards： | CGGCAAGAAGGCAGTCAAG |  |
|  | Reverse： | CCGTCGAGCACCGGTAGTAG |  |
| *PeWRKY45* | Forwards： | GACGGGTACAGGTGGAGGAA |  |
|  | Reverse： | AGTTTCTCGGGTTAGGGCTGTT |  |
| *PeWRKY51* | Forwards： | ACGGCAAGAAGATGGTCAAGA |  |
|  | Reverse： | TCGCTCGAGCAACGGTAGTA |  |
| *PeWRKY61* | Forwards： | CGCTGATGCCGGTTGAG |  |
|  | Reverse： | TGCACTCCTAGGATCAGCGTAGT |  |
| *PeWRKY83* | Forwards： | CCGCTCCTTTCGGTTCCT |  |
|  | Reverse： | GCCTGCCGCTGGTCCTA |  |
| *PeWRKY84* | Forwards： | CGGCAAGAAGGCAGTCAAG |  |
|  | Reverse： | CCGTCGAGCACCGGTAGTAG |  |
| *PeWRKY86* | Forwards： | TGTTGTGGTGACGACGTACGA |  |
|  | Reverse： | CAAAGTTGTCGCTGGCTCTCT |  |
| *TIP41* | Forwards： | AAAATCATTGTAGGCCATTGTCG |  |
|  | Reverse： | ACTAAATTAAGCCAGCGGGAGTG |  |
| *Tublin* | Forwards： | AGTGGTCGTACAACCGGTATT |  |
|  | Reverse: | GATGGCATGAGGAAGAGAGAA |  |
| *RD29A* | Forwards： | TGTGCCGACGGGATTTG |  |
|  | Reverse: | CTGATGCCTCACCGTATCCA |  |
| *RD29B* | Forwards: | TGTCTTCTGACCACACCAAACC |  |
|  | Reverse: | TGCCTCATGTCCGTAAGAGGTAT |  |
| *AtABF1* | Forwards: | CAACAACTTAGGCGGCGATAC |  |
|  | Reverse： | AAGACTGCCTCGCCAATGG |  |
| *AtNCED2* | Forwards: | CGGCGTCGGAGTAGCAAA |  |
|  | Reverse: | TCTGACATAGCTAAAAGCCGGTTA |  |
| *AtNCED3* | Forwards： | GTCGTCGTACCTGACCAGCAA |  |
|  | Reverse： | AGACCCACCGCGGATCAT |  |
| *AAO3* | Forwards： | CAACCGCATGCGCACTAG |  |
|  | Reverse： | GTCTTGCGGTTCAAAAACATCTT |  |
| *ABI1* | Forwards： | CCATGGCGGTTCTCAGGTA |  |
|  | Reverse： | CCTCCGCCAAAGCCAAA |  |
| *AtPP2CA* | Forwards： | CCGCTTGAAAACTGCCGTAA |  |
|  | Reverse： | CCGGTAACGTCGAGAGCACTAC |  |
|  |  | **Primers of constructing expression vector** |  |
| *PeWRKY83* | Forwards： | ATGTCGGGCGAGTTCCAG | BamHI |
|  | Reverse： | TCAAGGCTGGTTGCTGCT | EcoRI |
|  |  | **Primers for subcellular localization analysis** |  |
| **Gene** |  | **Primers sequences (5′→3′)** | **Restriction sites** |
| *PeWRKY83* | Forwards： | GGACTAGTATGTCGGGCGAGTTCCAG | *SpeI* |
|  | Reverse： | GCTCTAGAAGGCTGGTTGCTGCTGCT | *XbaI* |
|  |  | **Primers for transcription activity analysis** |  |
| **Gene** |  | **Primers sequences (5′→3′)** | **Restriction sites** |
| *PeWRKY83* |  | GGAATTCATGTCGGGCGAGTTCCAG | ***EcoRI*** |
|  |  | CGTCGACTCAAGGCTGGTTGCTGCT | ***BamHI*** |

**Table S5.** **List of 29 *VQ* genes identified in moso bamboo.**

| **Gene name** | **Sequence ID** |
| --- | --- |
| PeVQ1 | PH01000057G1670 |
| PeVQ2 | PH01000057G1670 |
| PeVQ3 | PH01000091G0990 |
| PeVQ4 | PH01000095G1340 |
| PeVQ5 | PH01000123G0310 |
| PeVQ6 | PH01000134G0110 |
| PeVQ7 | PH01000178G1300 |
| PeVQ8 | PH01000194G0080 |
| PeVQ9 | PH01000266G0360 |
| PeVQ10 | PH01000284G1000 |
| PeVQ11 | PH01000355G0340 |
| PeVQ12 | PH01000556G0810 |
| PeVQ13 | PH01000643G0660 |
| PeVQ14 | PH01000767G0790 |
| PeVQ15 | PH01000811G0090 |
| PeVQ16 | PH01001134G0540 |
| PeVQ17 | PH01001151G0360 |
| PeVQ18 | PH01001184G0450 |
| PeVQ19 | PH01001263G0090 |
| PeVQ20 | PH01001474G0380 |
| PeVQ21 | PH01001536G0420 |
| PeVQ22 | PH01001726G0080 |
| PeVQ23 | PH01002032G0210 |
| PeVQ24 | PH01002049G0250 |
| PeVQ25 | PH01003948G0070 |
| PeVQ26 | PH01004013G0020 |
| PeVQ27 | PH01004949G0070 |
| PeVQ28 | PH01007611G0010 |
| PeVQ29 | PH01211737G0010 |
